# Morpho-cultural, pathogenic, and genetic characterization of Indian isolates of *Macrophomina phaseolina* causing charcoal rot in soybean

**DOI:** 10.1016/j.heliyon.2025.e42035

**Published:** 2025-01-17

**Authors:** Laxman Singh Rajput, Sanjeev Kumar, Kriti Pathak, Palak Acharya, Divyanshu Goswami, Vennampally Nataraj, Maranna Shivakumar, Hemant Singh Maheshwari, Saloni Mandloi, Sapna Jaiswal, Asha Yadav, Raksha Vishwakarma

**Affiliations:** a ICAR-Indian Institute of Soybean Research, Indore, Madhya Pradesh, India; b ICAR-Central Arid Zone Research Institute, Jodhpur, Rajasthan, India; c Jawaharlal Nehru Krishi Vishwa Vidyalaya, Jabalpur, Madhya Pradesh, India; d Rajmata Vijayaraje Scindia Krishi Vishwa Vidyalaya, Gwalior, Madhya Pradesh, India; e The University of Groningen, Groningen, Netherlands

**Keywords:** Charcoal rot, Soybean, ITS, *Macrophomina phaseolina*, Morpho-cultural and pathogenic

## Abstract

*Macrophomina phaseolina*, a devastating soil and seed-borne fungus causing charcoal rot in soybean, poses a significant challenge to soybean production and breeding programs across all major soybean-growing regions of India. Fifty-five *M. phaseolina* isolates were collected from India's eight diverse soybean-growing agroecological regions. These isolates were examined for morpho-cultural, molecular, and pathogenic variability. All these isolates were pathogenic to the soybean and had significant variability for different Morpho-cultural characters. Principal component analysis (PCA) showed that most of Morpho-cultural traits are not having association with pathogenic traits. Cluster analysis showed that all these 55 isolates of *M. phaseolina* were classified into two major groups, and virulence characters did not separate based on origin. Group B showed more diversity and included the most virulent pathogen isolates. Phylogenetic analysis of the Internal Transcribed Spacer (ITS), a conserved rDNA region, revealed limited diversity among the 55 isolates. Irrespective of morpho-cultural and pathogenic characters, most isolates (n = 52) were clustered in a group. Pathogenic variability analysis has revealed region specific most virulent isolate from diverse agroecological regions of India. GGE biplot segregated the main effect of each component, cultivars (G), isolates (I), and G × I interactions with significant levels (*p < 0.001*). The virulence of isolates contributed 56.30 % of the total variation, followed by varieties (36.79 %) and G × I interaction (4.96 %). GGE biplot also provides information on two highly discriminative isolates. These isolates may be useful for screening genotypes and identifying quantitative trait loci (QTL) linked to soybean charcoal rot.

## Highlights

• Quantitative traits proved to be more effective for the characterization and classification of *Macrophomina* compared to qualitative traits.
• Phylogenetic analysis of the ITS revealed limited diversity among the 55 isolates.
• The isolates adapted to low temperatures are more virulent than those adapted to higher temperatures.
• The virulence of isolates (I) contributed 56.30 % of the total variation, followed by genotypes (36.79 %) and G × I interaction (4.96 %).
• MP-51 isolate was highly virulent while MP-50 and MP-52 isolates were highly discriminative.

## 1 Introduction

Soybean is a remarkable crop, contributing approximately 75 % of the total global protein concentrate for animal feed and 25 % to the global supply of edible oil [[1], [2], [3]]. The cultivation of soybeans has continuously faced major challenges due to various diseases in the world [4,5] and in India [6]. Among the diseases, charcoal rot of soybean is the most dangerous and destructive [7], contributing to a decline of 0.25 million tons of soybean yield globally each year, which is 4.2 % of total disease loss [5].

Soybean charcoal rot is caused by *M. phaseolina* and infects more than 500 crop species, including field crops, horticulture crops, and forestry [8,9]. Young [10] reported soybean charcoal rot from the United States of America for the first time. Later, the disease spread and was observed throughout the American and Asian continents [8]. The disease has increased over the past 30 years in the USA and different parts of the world [8]. The favourable epidemiological environmental conditions for soybean charcoal rot in Argentina during 2001–2002 led to a 100 % yield loss in some farmer fields [11]. Until 2004, charcoal rot disease in soybean was considered a minor disease in India. Unfortunately, alteration in climatic conditions, particularly prolonged high temperatures with drought spells during the reproductive growth stage of soybeans in major soybean growing areas of India, have led to a considerable increase in disease severity, causing considerable yield losses [6]. In Central India, a soybean charcoal rot epidemic was observed in 1997, which resulted in 80 % soybean yield loss in farmer fields [12].

Soybean charcoal rot is a major constraint for all major soybean-growing countries [13]. High temperatures and drought-like situations create the most favourable epidemiological environmental conditions for soybean charcoal rot, leading to complete crop failure [2,3]. Under unfavourable epidemiological environmental conditions, significant yield losses ranging from 6 % to 33 % in susceptible cultivars were observed [14]. In India, favourable epidemiological environmental conditions caused 77 % of yield loss observed due to charcoal rot disease [15].

Recently, the cropping pattern of central India has changed from soybean followed by wheat to soybean followed by chickpea [16]. Due to a similar range of plant pathogens, this change could potentially worsen soil-borne diseases, particularly charcoal rot in central India [3]. Soybean mega-varieties in India, such as Jabalpur Soybean (JS) 335, JS 93-05, and JS 95-60 have become susceptible to charcoal rot [2]. The main factor for catastrophic disease development is the unavailability of resistant cultivars [17]. The absence of resistance in the cultivated varieties may be linked to variability in the *M. phaseolina* population and the multi-dimensional mode of pathogenicity [8].

Limited plant protection options are available to manage soybean charcoal rot. Farmers are advised to grow moderately susceptible cultivars and avoid stress in plants by maintaining recommended populations of plants, avoiding crop injury, and applying irrigation at drought stress during reproductive growth stages [3,14,18,19]. Other management strategies for soybean charcoal rot are the use of seed dressing fungicides [20,21], foliar application of systemic fungicides [22], and soil and seed application of antagonists [23,24]. However, these management strategies have found limited success in reducing disease severity. Various researchers identified that different isolates showed variations in sensitivity to different fungicides and bio-control agents [25,26].

The soybean charcoal rot pathogen, *M. phaseolina,* shows an immense variation in morphological [7,[27], [28], [29], [30], [31]], pathogenic [7,[29], [30], [31], [32], [33]] and genetic characteristics [[34], [35], [36]], that enhanced its phytopathological adaptability to diverse agro-ecological climatic conditions.

In previous works, the diversity of soybean charcoal rot pathogen has not been extensively studied in India, and most of them were limited to region-specific studies [7,31]. Understanding the morpho-cultural, pathogenic, and genetic diversity of *M. phaseolina* is necessary for developing efficient disease management modules, improving disease screening techniques by identification of regional specific virulent isolates, allowing the development of stable resistance genotypes, and enhancing a better understanding of disease epidemiology [9,37].

The present investigation aims to address this research gap by characterizing morpho-cultural traits, pathogenicity, and genetic diversity in 55 isolates of *M. phaseolina* collected from soybean-growing seven agroecological zones in India. The findings from this investigation may assist plant breeders in identifying stable resistant genotypes and aid plant pathologists in evaluating various systemic fungicides and bioagents to develop region-specific disease management modules. Our investigational approach also resulted in identification of regional specific virulent isolates.

## 2 Materials and methods

### 2.1 Collection and isolation of *M. phaseolina* isolates from major soybean cultivation areas of India

Comprehensive surveys were undertaken to collect *M. phaseolina* isolates from different soybean-growing regions of India, spanning the cropping seasons from 2018 to 2020 (Table 1), encompassing various soil types such as alluvium, red, black, and laterite. Most collections (n = 38 isolates) were obtained from India's central and western plateau and hilly agro-ecological regions. These regions predominantly encompass the soybean cultivation areas [7]. The infected stem portion was collected in butter paper bags, labeled properly, and subsequently preserved in cool buckets to avoid the risk of secondary microbial invasion.

**Table 1:** List of *M.* phaseolina isolates collected from soybean growing area of India.

Isolate number	Place of collection	District	State	Soil	Agro-ecological region	Accession number
MP 1	Indore	Indore	Madhya Pradesh	Medium black	Western plateau and hills region	MZ901367
MP 2	Hatod	Indore	Madhya Pradesh	Medium black	Western plateau and hills region	MT126642
MP 3	Rau	Indore	Madhya Pradesh	Medium black	Western plateau and hills region	MT127374
MP 4	Sanwer	Indore	Madhya Pradesh	Medium black	Western plateau and hills region	MT127378
MP 5	Depalpur	Indore	Madhya Pradesh	Medium black	Western plateau and hills region	OR467501
MP 6	Ratlam	Ratlam	Madhya Pradesh	Medium black	Western plateau and hills region	MT122794
MP 7	Ujjain	Ujjain	Madhya Pradesh	Medium black	Western plateau and hills region	MT126641
MP 8	Dewas	Dewas	Madhya Pradesh	Medium black	Western plateau and hills region	OP146091
MP 9	Sehore	Sehore	Madhya Pradesh	Medium black	Central plateau and hills region	OP143852
MP 10	Sonkatch	Sehore	Madhya Pradesh	Medium black	Central plateau and hills region	MT127393
MP 11	Kothari	Sehore	Madhya Pradesh	Medium black	Central plateau and hills region	OP164542
MP 12	Neponiya Sikka	Sehore	Madhya Pradesh	Medium black	Central plateau and hills region	OR469963
MP 13	Kheri	Sehore	Madhya Pradesh	Medium black	Central plateau and hills region	OP146137
MP 14	Mandsaur	Mandsaur	Madhya Pradesh	Medium black	Western plateau and hills region	MT126638
MP 15	Jhabua	Jhabua	Madhya Pradesh	Medium black	Western plateau and hills region	OP135608
MP 16	Piploddh	Khandwa	Madhya Pradesh	Medium black	Western plateau and hills region	OP164421
MP 17	Borkheda	Khandwa	Madhya Pradesh	Medium black	Western plateau and hills region	OR469962
MP 18	Morena	Morena	Madhya Pradesh	Alluvial light	Central plateau and hills region	OP146103
MP 19	Jabalpur	Jabalpur	Madhya Pradesh	Medium Black	Central plateau and hills region	OR467498
MP 20	Betul	Betul	Madhya Pradesh	Shallow black	Central plateau and hills region	MT126640
MP 21	Amravati	Amravati	Maharashtra	Black	Western plateau and hills region	MT126643
MP 22	Yavatmal	Amravati	Maharashtra	Black	Western plateau and hills region	MT127373
MP 23	Wardha	Wardha	Maharashtra	Black	Western plateau and hills region	MT127375
MP 24	Parbhani	Parbhani	Maharashtra	Black	Western plateau and hills region	OR469960
MP 25	Nagpur	Nagpur	Maharashtra	Black	Western plateau and hills region	OP146134
MP 26	Akola	Akola	Maharashtra	Black	Western plateau and hills region	OP146113
MP 27	Buldhana	Buldhana	Maharashtra	Black	Western plateau and hills region	OP146134
MP 28	Pune	Pune	Maharashtra	Black	Western plateau and hills region	MT127379
MP 29	Amravati	Amravati	Maharashtra	Black	Western plateau and hills region	OP164532
MP 30	Kasbe-digraj	Sangli	Maharashtra	Black	Western plateau and hills region	MT127377
MP 31	Amreli	Amreli	Gujrat	Black	Gujarat plains and hills region	MT127381
MP 32	Bhavnagar	Bhavnagar	Gujrat	Black	Gujarat plains and hills region	MT119461
MP 33	Dahod	Dahod	Gujrat	Black	Gujarat plains and hills region	MT127382
MP 34	Navsari	Navsari	Gujrat	Alluvial	Gujarat plains and hills region	MT127383
MP 35	Banswada	Banswada	Rajasthan	Black	Central plateau and hills region	MT126639
MP 36	Banswada	Banswada	Rajasthan	Black	Central plateau and hills region	MT127384
MP 37	Dungarpur	Dungarpur	Rajasthan	Black	Central plateau and hills region	OP146441
MP 38	Kota	Kota	Rajasthan	Alluvial	Central plateau and hills region	MT127385
MP 39	Umedganaj	Kota	Rajasthan	Alluvial	Central plateau and hills region	OP143965
MP 40	Udaipur	Udaipur	Rajasthan	Alluvial	Central plateau and hills region	MT127386
MP 41	Chittorgarh	Chittorgarh	Rajasthan	Alluvial	Central plateau and hills region	MT127387
MP 42	Baran	Baran	Rajasthan	Alluvial	Central plateau and hills region	MT127397
MP 43	Adilabad	Adilabad	Telangana	Black	Southern plateau and hills region	MT127380
MP 44	Dharwad	Dharwad	Karnataka	Red	Southern plateau and hills region	MT127388
MP 45	Belgavi	Belgavi	Karnataka	Red	Southern plateau and hills region	OP146136
MP 46	Raipur	Raipur	Chhattisgarh	Red	Eastern plateau and hills region	MT127389
MP 47	Varanasi	Varanasi	Uttar Pradesh	Alluvial	Middle Gangetic Plains region	MT127391
MP 48	Patna	Patna	Bihar	Alluvial	Middle Gangetic Plains region	MT127392
MP 49	Ranchi	Ranchi	Jharkhand	Laterite	Eastern plateau and hills region	OP146443
MP 50	Imphal	Imphal	Manipur	Laterite	Eastern Himalayan Region	MT127394
MP 51	Medziphema	Dimapur	Nagaland	Laterite	Eastern Himalayan Region	MT127395
MP 52	Ludhiana	Ludhiana	Punjab	Alluvial	Trans Gangetic plains region	MT127396
MP 53	Khurampur	Jalandhar	Punjab	Alluvial	Trans Gangetic Plains region	OP164540
MP 54	New Delhi	New Delhi	New Delhi	Alluvial	Trans Gangetic Plains region	OR467499
MP 55	Coimbatore	Coimbatore	Tamil Nadu	Red	Southern plateau and hills region	OR469961

Fifty-five isolates of *M. phaseolina* were characterized from 154 diseased samples based on morpho-cultural and pathogenic variability evaluated under controlled glasshouse conditions. The infected stem's small bits (5–6 mm) were subjected to surface sterilization using 0.1 % sodium hypochlorite solution for 45 s, then rinsed thrice with sterile distilled water. These segments were transferred onto Petri plates with potato dextrose agar (PDA) medium (Hi Media, India). After four days of incubation at 25 ± 2 °C in BOD, the Petri plates exhibited cottony mycelia growth of *M. phaseolina* [38]. The single sclerotial body was harvested from Petri plates with the help of a compound microscope for *M. phaseolina* and placed in PDA plates acidified with 0.2 % lactic acid to obtain a pure culture. The pure culture of the fungus was preserved at 5 °C for future studies and was sub-cultured once a month. Every year, the pathogen virulence was maintained through the pot culture of the host (*Glycine* max (L.) Merr. cultivar JS 95-60, and the pathogen isolated from infected tissue showed characteristic symptoms [39].

### 2.2 Morpho-cultural variability of *M. phaseolina* isolates

The isolates of *M. phaseolina* were examined for radial growth, growth rate, constriction at the base, colony colour, colony margin, aerial growth, dry weight, number of micro-sclerotial bodies per cm^2^, size of microsclerotia, mycelial cell size, and hyphal width. In adherence to strict aseptic protocols, 4 mm of pure culture of mycelium discs were obtained from 9-day-old cultures of various *M. phaseolina* isolates. These mycelial discs were centrally placed in a sterilized 90 mm diameter Petri plate containing PDA to study cultural and morphological characters. The culture plates of each isolate were kept in three replications and incubated and at a controlled temperature of 27 ± 2 °C in BOD. Further, cultural and morphological parameters were estimated.

The radial growth was assessed by measuring the diameter of mycelium after the second and third days of incubation. Based on two days of radial growth, isolates were divided into four categories: excellent (>7 cm), good (5–7 cm), moderate (4–5 cm), and slow (2–4 cm). The time required for full plate growth of isolate was also determined [40].

The surface of mycelium was visually examined and classified into three types, *i.e.*, compact (CMP), fluffy (iffy), and cottony growth (ctn). The colony colour was assessed utilizing Munsell's soil colour chart [41]. The culture plate was placed alongside a reference colour card, and the colony colour was observed from the both the side of the culture plate. Each isolate was characterized according to the appearance of the margin, whether regular or irregular. The presence and absence of aerial growth were visualized in 9 days of the culture plate.

The dry weight of the isolate was obtained from each *M. phaseolina* isolate. The isolates of *M. phaseolina* were grown in 100 ml of potato dextrose broth for 9 days at 27 ± 2 °C. The mycelium was carefully harvested in pre-weighed Whatman filter paper number 114 from the culture medium and washed to remove contaminants or residual media. The mycelium was dried thoroughly at 70 °C ± 2 °C for 4 days using a hot air oven to remove all moisture content. After complete drying, the dry weight of mycelium was measured [42].

Morphological studies were conducted with four-day-old pure cultures of *M. phaseolina* isolates. Mycelium harvested from cultures of four days old Petri plates was stained with aniline blue (0.5 %) in lacto-phenol solution. Afterward, a glass cover slip was carefully placed to ensure comprehensive coverage. The characters of hypha and microsclerotia were visualized in twin software of Leica compound (Model DMRBE) microscope attached with photomicrography unit (DFC295) 40× and 10× eyepiece objectives. The observations were made at 40× magnification, and accompanying images were captured to measure morphological characters. Visual observations were also recorded as the presence or absence of constriction at the branching base, employing the procedure outlined by Ref. [43].

The length and width of 20 microsclerotial bodies were measured in replicates*.* Mycelial cell size and hyphal width were quantified in each replication by averaging 20 hyphae from the mycelial mat within the microscopic field. The number of microsclerotial bodies per cm^2^ was determined through a hemocytometer in a 4-day-old pure culture of each isolate of *M. phaseolina*. All the data was statistically analyzed through R Studio (https://posit.co/download/rstudio-desktop) [44].

### 2.3 Pathogenic variability of *M. phaseolina* isolates

To check the pathogenicity, two susceptible soybean cultivars (JS 95-60) and (Shivalik) and one moderate resistance cultivar at field condition (JS 20–98) were selected for the identification of virulent isolate of *M. phaseolina* and to study the interaction between the isolates of *M. phaseolina* and different cultivars of soybean. Untreated seeds of all three cultivars were sowed in pots, containing sterilized sand, soil, and vermicompost in equal proportion. The experiment was setup in a glasshouse under optimal conditions (30 ± 2 °C day/21 ± 2 °C night) to promote disease expression. Healthy, uniformly grown plants were selected for inoculation in each pot, with each replication consisting of two pots. The experiment followed a completely randomized design with three replications per treatment. Each isolate was inoculated across all three cultivars, with each combination treated as a separate experimental treatment. A precise incision was made 25 mm above the unifoliate node in 15-day-old plants using a sterilized razor blade. A four-day-old culture of *M. phaseolina* was collected in a sterile 20 μl pipette tip (Fisher Scientific), through insertion into the culture plate. The entire culture was placed inside a sterile 20 μl pipette tip, which was then carefully positioned at the apex of the blunted end of the soybean stem. The pathogen culture was inserted precisely to minimize the risk of disease escape [3,38]. Inoculation leads to developed lesion on stem. The pipette tips were removed from each plant and discarded two days after inoculation. Disease progression was assessed by measuring lesion length (linear stem necrosis) in centimeters using a ruler at 2, 7 and 14 days after inoculation (DAI). Infection caused by *M. phaseolin* on apex of stem leads to frequent rotting and subsequent detachment of stem therefore lesion length was measured from unifoliolate node and added to 25 mm to determine the total length of necrosis. If the necrosis did not reach the unifoliolate node, the distance from the node to the necrosis was measured and subtracted from the initial 25 mm. The area under the disease progress curve (AUDPC) according to the formula $AUDPC=\sum_{t=1}^{n-1}\left(\left(L_{i}+L_{t_{i+1}}\right)/2\right)\times \left(t_{i+1}-t_{i}\right)$, where Li was lesion length (cm) at the ith observation, t_i_ was the time (days) in ith observation, and n was the total number of observations [45].

### 2.4 Molecular variability of *M. phaseolina* isolates

The pure culture of *M. phaseolina* was multiplied in Potato Dextrose Broth for five days at 27 °C ± 2 °C with shaking at 100 rpm in BOD. The mycelium was harvested by filtration using a sterilized Whatman filter paper number, followed by careful rinsing with sterilized distilled water and was then cautiously dried between layers of tissue. Subsequently, the harvested mycelium was taken to extract total genomic DNA by utilizing the extraction and purification kit (HiPurA™ fungal DNA purification kit; HiMedia) following the manufacturer's instructions [46]. Quantity and quality of DNA were ensured through a Nano-drop Spectrophotometer.

The ITS region of the rDNA of *M. phaseolina* isolates was amplified [47]. Each PCR reaction mixture (50 μL) contained 50 ng of genomic DNA, 2 X dream taq green PCR master mix (Genetix Biotech Asia), and 10 Pmol of each primer. The PCR reaction was performed with 57 °C of annealing in a thermocycler. Agarose gel electrophoresis stained with ethidium bromide was performed to separate the PCR product and a 1 kb DNA ladder for 1 h at 80 V and visualized in the gel documentation system. The amplicons were excised from the gel and purified. The purified amplicon was sequenced by the BigDye™ terminator v3.1 cycle sequencing method (Eurofins Genomics India Pvt. Ltd). The data obtained from the partial ITS-5.8S rDNA sequence of 55 *M. phaseolina* isolates underwent manual inspection and editing utilizing Chromas software, and alignment of the sequences was conducted using Clustal-W [48]. The sequences were deposited in NCBI GenBank with the accession number provided in Table 1. Phylogenetic analysis used different sequences to identify sequence similarity among the *M. phaseolina* isolates. Multiple alignments were performed through the MUSCLE algorithm of MEGA 11.1 software, and a Maximum likelihood phylogenetic tree was constructed with confidence intervals of 1000 bootstrap replicates.

### 2.5 Statistical analysis

Analysis of variance (ANOVA) and multiple comparison tests based on the Least Significant Difference (LSD) test were done through the 'Agricolae' package of R studio software version R 4.2.3 [44] (https://posit.co/download/rstudio-desktop). The Shapiro-Wilk test was used to assess the normality of the data, with a p-value greater than 0.05 indicating a normal distribution [49]. All the data were normally distributed. PCA was performed using the R package 'Factoextra' to identify key morpho-cultural and pathogenic traits for *M. phaseolina* characterization and classification [50,51]. Quantitative traits were used in their original form, while qualitative traits were converted into numerical values using a dummy coding system to facilitate PCA analysis. Morpho-cultural and pathogenic traits radial growth at 2 days (RG), radial growth at 3 days (RG2), growth rate (GR), dry weight (DW), number of sclerotia (NS), mycelial cell size (CS), hyphal width (HW), width of micro-sclerotia (SSB), length of micro-sclerotia (SSL), AUDPC on JS 95-60 (A1), AUDPC on Shivalik (A2) and AUDPC on JS 20–98 (A3) were consider as Quantitative traits for PCA. Morpho-cultural traits constriction at the base (CB), upper surface colony colour (USC), lower surface colony colour (LSC), colony surface (CoS), margin of colony (MC) and aerial growth (AG) were consider as qualitative traits for PCA. GGE biplot analysis was conducted using the R package 'metan' to illustrate the interactions between isolates and cultivars, identify host specificity, and determine the most discriminative isolates [52].

## 3 Results

### 3.1 Morpho-cultural variability of *M. phaseolina* isolates

The soybean plant samples exhibiting typical diseased symptoms such as leaf drooping and silver to light-gray discolouration on lower stem sections were collected from surveyed regions. A total of 154 samples were collected and subjected to isolation, and 55 distinct isolates of *M. phaseolina* were obtained. These isolates were derived from eight distinct agroecological zones associated with 13 states and two union territories of the soybean cultivation area of India. Initial identification of these isolates was performed based on morpho-cultural traits, including colony colour, texture, margin characteristics, and the production of microsclerotia. Morpho-cultural variability was observed irrespective of the agro-climatic zone; however, radial growth, number of microsclerotial bodies per cm^2^, size of microsclerotia, and hyphal width at optimum temperature showed a significant variation.

Significant differences were observed in the radial growth of 2 days after incubation (*F= 55.65; df= 54, 108; P < 0.001*) and 3 days after incubation (*F= 194.52; df= 54, 108; P < 0.001*) among 55 isolates of *M. phaseolina* obtained from 8 different agroecological regions of India (Table S1). The average radial growths of 55 isolates of *M. phaseolina* ranged from 2.67 to 8.08 cm observed 2 days after incubation. Maximum radial growth after 2 days of incubation was 8.07 cm observed with isolate MP-21 (Amravati), which did not show significant difference with isolates MP-2 (Hatod), MP-4 (Sanwer), MP-8 (Dewas), MP-54 (New Delhi) and MP-11 (Kothari), whereas, isolates MP-23 and MP-24 showed the minimum radial growths. The average radial growths of 55 isolates of *M. phaseolina* ranged from 4.25 to 8.40 cm observed 3 days after incubation. Maximum radial growth after 3 days of incubation 8.40 cm was observed with isolates MP-2 (Hatod), MP-3 (Rau), MP-6 (Ratlam), MP-7 (Ujjain), MP-15 (Jhabua), MP-21 (Amravati), MP-35 (Banswada), which did not show significant difference with isolates MP-14 (Mandsaur), MP-19 (Jabalpur), MP-1 (Indore) and MP-22 (Yavatmal), whereas, minimum radial growths was exhibited by isolates MP-23 and MP-24. Among the 55 isolates, ten displayed a radial growth exceeding 7.00 cm and were categorized as excellent growers. Conversely, 2 isolates exhibited growth below 4.00 cm and were defined as slow growers. A total of 36 isolates exhibited good growth, while 7 isolates exhibited moderate growth. All these isolates did not show a significant difference in dry mycelia weight.

These isolates differed in their morpho-cultural characteristics, such as upper and lower colony colour, and were categorized into four groups: black, dark gray, gray, and light gray. A total of 63.63 % of the isolates showed compact growth, whereas the remaining isolates showed fluffy growth. A total of 83.63 % of isolates showed a typical greyish shade for the upper colony colour, whereas 50.90 % of isolates showed a typical greyish shade for the lower colony colour. Significant variation was not observed for the margin of colony and aerial growth; most of the isolates showed a regular margin of colony (89.09 %) and aerial growth (80.00 %).

Significant differences were observed in a number of microsclerotia (*F= 5.64; df= 54, 108; P < 0.001*), length (*F= 10.89; df= 54, 108; P < 0.001*) and microsclerotia width (*F= 19.36; df= 54, 108; P < 0.001*), mycelia cell size (*F= 8.05; df= 54, 108; P < 0.001*) and hyphal width (*F= 11.35; df= 54, 108; P < 0.001*) among different isolates of *M. phaseolina* (Table S2). The average number of microsclerotia of *M. phaseolina* ranged from 75000 to 1563750. Among these isolates, MP-38 (Kota) produced a maximum number of microsclerotia, which was significantly higher (*P < 0.001*) than other isolates, whereas a minimum number of microsclerotia was produced by MP-20 (Betul). The average size of microsclerotia of *M. phaseolina* ranged from 41.26 to 314.65 μm (length) and 36.48–304.20 (width) μm. The largest microsclerotia produced by isolate MP-32 (Bhavnagar) was 314.65 μm (length) × 304.20 (width) μm, whereas the smallest microsclerotia produced by MP-10 (Sehore) of size 41.26 × 36.48 (width) μm. The average size of the mycelial cells of 55 isolates of *M. phaseolina* ranged from 17.06 to 59.32 μm. The maximum size of the mycelial cells was observed in isolates MP-26 (Akola) and MP-2 (Hatod), while isolates MP-53 (Khurampur) showed the minimum mycelial cell size. The average size of the hyphal width of *M. phaseolina* ranged from 3.82 to 14.46 μm. The maximum size of hyphal width was observed with isolates MP-26 (Akola), while isolating MP-51 (Medziphema) showed the minimum size of hyphal width.

### 3.2 Pathogenic variability of *M. phaseolina* isolates

All the isolates of *M. phaseolina* showed typical symptoms in pathogenicity studies. Charcoal rot symptoms were not observed on seedlings in control pots without inoculum. Seventy-five seedlings were taken for each cultivar (25/each replication), a total of 3 replications for each isolate. Nearly every isolate showed symptoms of charcoal rot disease in each soybean cultivar (Table S3).

Our results revealed that a significant difference was observed in AUDPC for isolates (I), cultivars (G), and G × I (Table S4). The significant G × I interaction concluded that virulence in isolates and resistance in varieties varied with the environment. Our results also showed that the variation in the virulence of *M. phaseolina* was primarily influenced by the isolates (I), contributing 56.30 % of the variation, followed by cultivars (G) at 36.79 %, and the G × I interaction at 4.96 %, indicating that most of the variation for disease reaction was depend upon virulence of isolates. Isolate MP-51 (Medziphema) was found in most virulent isolates with a mean AUDPC of 130.54, a significantly higher (*P < 0.001*) AUDPC than other isolates. The least virulent isolate, MP 15 (Jhabua), had a mean AUDPC of 3.52. Notably, this isolate exhibited the lowest AUDPC across all three varieties: JS 95-60 (2.44), Shivalik (2.23), and JS 20–98 (5.90). The variety JS 20–98 provided a maximum AUDPC of 50.50, significantly higher (*P < 0.001*) than other varieties JS 95-60 (35.82) and Shivalik (39.77). The isolate MP-52 (Ludhiana) produced the highest AUDPC, 134.16 on Shivalik, which was significantly higher (*P < 0.001*) than other isolates on varieties except MP 51 (Medziphema) on Shivalik, MP 51(Medziphema) on 95-60, MP 51(Medziphema) on 20–98, MP 51 (Medziphema) on Shivalik, MP 36 (Banswada) on JS 20–98; MP 8 (Dewas) on JS 20–98; MP 50 (Imphal) on JS 20–98; MP 39 (Kota) on JS 20–98; MP 34 (Navsari) on JS 20–98.

Significant differences were observed in AUDPC for varieties JS 95-60 (*F= 6.25; df= 54, 108; P < 0.001*), Shivalik (*F= 6.80; df= 54, 108; P < 0.001*), and JS 20–98 (*F= 11.31; df= 54, 108; P < 0.001*) among the different isolates of *M. phaseolina* (Table S3). The assessment of average AUDPC is used to categorize isolates into distinct groups using the least significant difference test. The average AUDPC of 55 isolates of *M. phaseolina* ranged from 2.44 to 131.46 for JS 95-60, 2.23–134.15 for Shivalik and 5.90–126.69 for JS 20–98. Maximum AUDPC in variety JS 95-60 was observed with MP-51(Medziphema), which showed significantly higher (*P < 0.001*) AUDPC except for isolates MP 19 (Jabalpur), MP 36 (Banswada), MP 52 (Ludhiana). In contrast, maximum AUDPC in variety Shivalik was observed with MP-52 (Ludhiana), which showed significantly higher (*P < 0.001*) AUDPC except for isolates, MP 12 (Neponiya Sikka), MP 13 (Kheri), MP 21(Amravati), MP 29 (Amravati), MP 30 (Kasbedigraj), MP 34 (Navsari), MP 36 (Banswada), MP 39 (Umedganaj), MP 40 (Udaipur) MP 51 (Medziphema). Similarly, maximum AUDPC in variety JS 20–98 was observed with MP-51(Medziphema) which showed significant higher AUDPC except for isolates MP 8 (Dewas), MP 11 (Kothari), MP 12 (Neponiya Sikka), MP 17 (Borkheda), MP 21 (Amravati), MP 24 (Parbhani), MP 29 (Amravati), MP 30 (Kasbedigraj), MP 36 (Banswada), MP 39 (Umedganaj), MP 44 (Dharwad) and MP 50 (Imphal).

Cultivars (G)-by-isolates (I) interaction GGE biplot was also constructed to known pathogenic variability among the isolates. GGE biplot was organized into four quadrants by two primary principal component axes. The first two principal components (PCs) explained 100.0 % of the host-isolate variation (Fig. 1), suggesting that the biplot effectively concludes the complex interactions between different genotypes and isolates. Specifically, PC1 accounts for 70.64 % of the variation, while PC2 accounts for 29.36 %. The horizontal axis, PC1, represents the first principal component of MP isolates, and the vertical axis, PC2, represents the second principal component for soybean varieties. GGE biplot for ranking environment illustrates the relative susceptibility of various varieties to isolates, using the proximity to the 'ideal' genotype - a standard representing the maximum susceptibility as a measure. Variety JS 20–98 was near this ideal genotype and represented highly susceptible, whereas variety JS 95-60 and Shivalik had lower susceptibility to all the isolates.

**Fig. 1:**
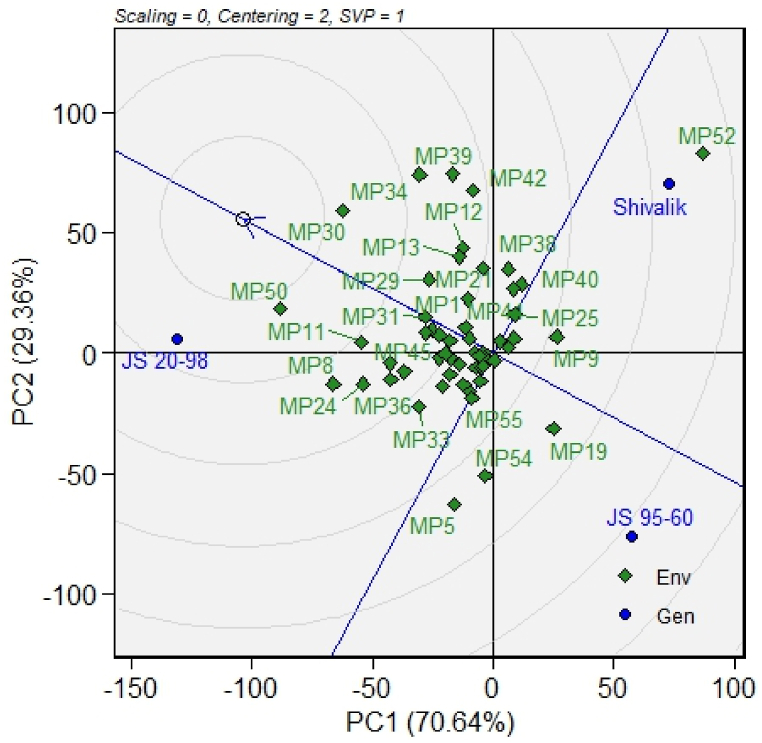
GGE biplot for ranking cultivars (JS 95-60, JS 20–98, and Shivalik) focused scaling for comparison of the cultivars with the ideal cultivars, based on mean and stability to an AUDPC of 55 isolates of *M. phaseolina* (MP1 to MP55) collected from major soybean growing agroecological zone of India. Details of isolates (MP 1 to MP 55) were presented in Table 1 and AUDPC of 55 isolates of *M. phaseolina* was presented in Table S4.

The GGE biplot analysis for which won where/what indicated that based on the interactions observed between varieties and winning isolates, along with the different degrees of virulence and host resistance, the analysis can be effectively partitioned into three sections, each representing different wining isolates in a variety (Fig. 2). Most isolates did not win any genotypes as they were inside the triangle. Variety JS 20–98 was winning 12 isolates collected from different agro-ecological zones i.e, MP-50 (Imphal), MP-30 (Kasbedigraj), MP-34 (Navsari), MP-29 (Amravti), MP-12 (Neponiya Sikka), MP-13 (Kheri), MP-36 (Banswara), MP-33 (Dhahod), MP-45 (Belgavi), MP-24 (Parbhani), MP-8 (Dewas). Variety JS 95-60 was won by two isolates, MP-54 (New Delhi) and MP-5 (Depalpur), whereas variety Shivalik also won by two isolates, MP-49 (Ranchi) and MP 39 (Umedganaj). The majority of the isolates were non-discriminatory, as they were located near the center of the GGE plot. The isolates MP-52 (Ludhiana) and MP-50 (Imphal) were the most discriminative for all the varieties as they were far from the plot's center. The most virulent isolate, MP-51 (Medziphema), with the highest mean of AUDPC of 130.54, and the least virulent MP 15 (Jhabua), with the lowest mean of AUDPC of 3.52, was found near the centre, indicating the least discriminative isolates.

**Fig. 2:**
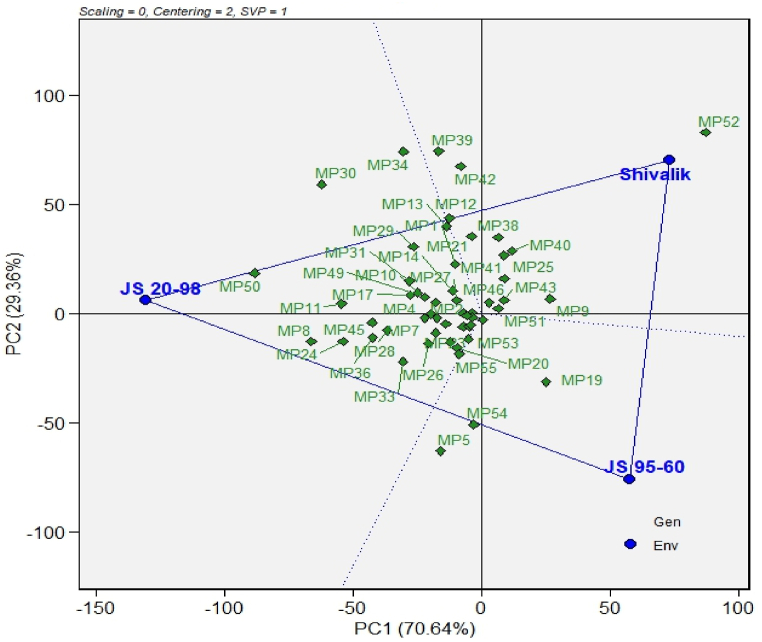
A triangle view of the GGE biplot showing which isolates of *Macrophomina phaseolina* (MP1 to MP55) from major soybean-growing agroecological zones of India had higher AUDPC values against the soybean cultivars JS 95-60, JS 20–98, and Shivalik. Details of isolates (MP 1 to MP 55) were presented in Table 1 and AUDPC of 55 isolates of *M. phaseolina* was presented in Table S4. The isolates positioned closest to the cultivars, but located outside the triangle, are considered the winning isolates. The isolates located within the triangle did not exhibit dominance over any cultivars.

Cluster analysis was also conducted on 3 pathogenic characters. The ward method of culture analysis was used, forming three major groups, labeled as cluster I, cluster II, and cluster III (Supp Fig. 2). The isolates were not clearly distinguished based on diverse climates by the cluster analysis of pathogenic traits. The isolates with lower virulence were clustered in cluster III, whereas isolates with higher virulence were grouped separately in clusters I and II.

### 3.3 Combined morpho-cultural and pathogenic variability of *M. phaseolina* isolates

Combined morpho-cultural and pathogenic variability was obtained from PCA and cluster analysis. PCA was performed to identify the key independent variables and quantify the total variance explained by select components. The PCA was conducted using 15 morpho-cultural traits and 3 pathogenic traits. The analysis revealed principal components and their corresponding proportions of explained variation, as determined by eigenvectors (Table 2).

**Table 2:** Eigen vector values for morpho-cultural and pathogenic traits of isolates of *M. phaseolina*.

Eigen vector	PC 1	PC 2
Radial Growth 2 days (RG)	−0.14	0.53
Radial Growth 3 days (RG2)	−0.11	0.51
Growth rate (GR)	−0.15	0.55
Constriction at the base (CB)	−0.05	0.08
Upper surface colony colour (USC)	−0.22	−0.03
Lower surface colony colour (LSC)	−0.07	0.00
Colony surface (CoS)	−0.21	−0.02
Dry weight (DW)	−0.15	−0.16
Number of sclerotial (NS)	−0.09	0.13
Mycelial cell size (CS)	0.17	0.02
Hyphal width (HW)	0.10	−0.08
Width of micro-sclerotia (SSB)	0.20	0.19
Length of micro-sclerotia (SSL)	0.22	0.15
Margin of colony (MC)	0.13	−0.05
Aerial growth (AG)	−0.26	0.04
AUDPC on JS 95-60 (A1)	−0.46	−0.12
AUDPC on Shivalik (A2)	−0.44	−0.06
AUDPC on JS 20–98 (A3)	−0.45	−0.13

The first principal components (PC1 and PC2) mostly captured the maximum variation in radial growth and growth rate of morpho-cultural characters alongside pathogenic characters such as AUDPC in different varieties. This indicated that quantitative characters captured more variance than qualitative characters. The first two principal components (PC1 and PC2) mostly captured the maximum variation of isolates, i.e., MP 23 (Wardha), MP 24 (Parbhani), MP 21 (Amravati), MP 36 (Banswada), MP 48 (Patna), MP 17 (Borkheda).

**Fig. 3:**
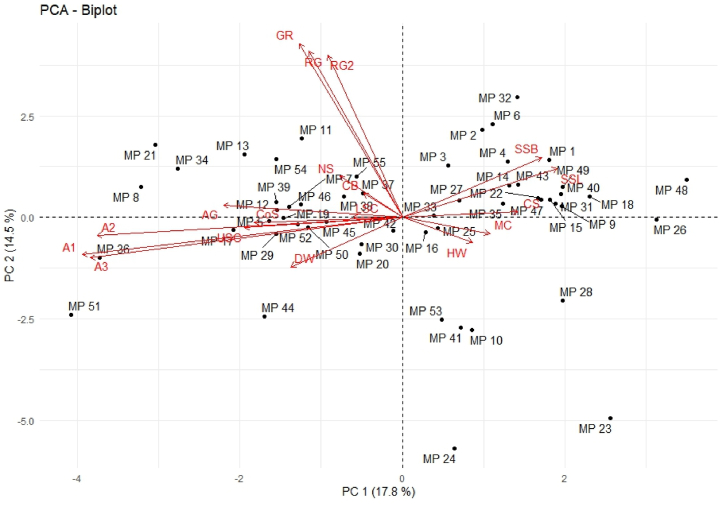
PCA for 15 morpho-cultural traits and 3 pathogenic traits of 55 isolates of *M. phaseolina* collected from major soybean growing agroecological zone of India. Details of isolates (MP 1 to MP 55) are presented in Table 1, and morpho-cultural and pathogenic characters are in Table 2 and Tables S1–S3. Morpho-cultural traits radial growth at 2 days (RG), radial growth at 3 days (RG2), growth rate (GR), dry weight (DW), number of sclerotia (NS), mycelial cell size (CS), hyphal width (HW), width of micro-sclerotia (SSB), length of micro-sclerotia (SSL), constriction at the base (CB), upper surface colony colour (USC), lower surface colony colour (LSC), colony surface (CoS), margin of colony (MC) and aerial growth (AG); and pathogenic traits AUDPC on JS 95-60 (A1), AUDPC on Shivalik (A2) and AUDPC on JS 20–98 (A3) were consider for PCA.

The first two principal components (PCs) explained 32.34 % of the total variation (Table S5). A total of 7 dimensions has eigenvalue >1.0 and capture 77.43 % of the total variation in different morpho-cultural and pathogenic characters. PCA analysis grouped isolates into four clusters (Fig. 3). Cluster I included low virulent isolates (MP 15, MP 18, and MP 22) positively associated with micro-sclerotial size and colony surface. Cluster IV included high virulent isolates (MP-51 and MP-36), positively associated with AUDPC on different verities, mycelia's dry weight, and mycelia's colour. Isolates of cluster III were positively associated with mycelia and aerial growth, whereas isolates of cluster II were positively associated with cell size and hyphal width. AUDPC on different verities showed a negative association with the size of micro-sclerotia and colony surface, whereas it showed a positive association with the colour of the colony. AUDPC on different verieties didn't show any association with other morpho-cultural characters, including a number of micro-sclerotia.

**Fig. 4:**
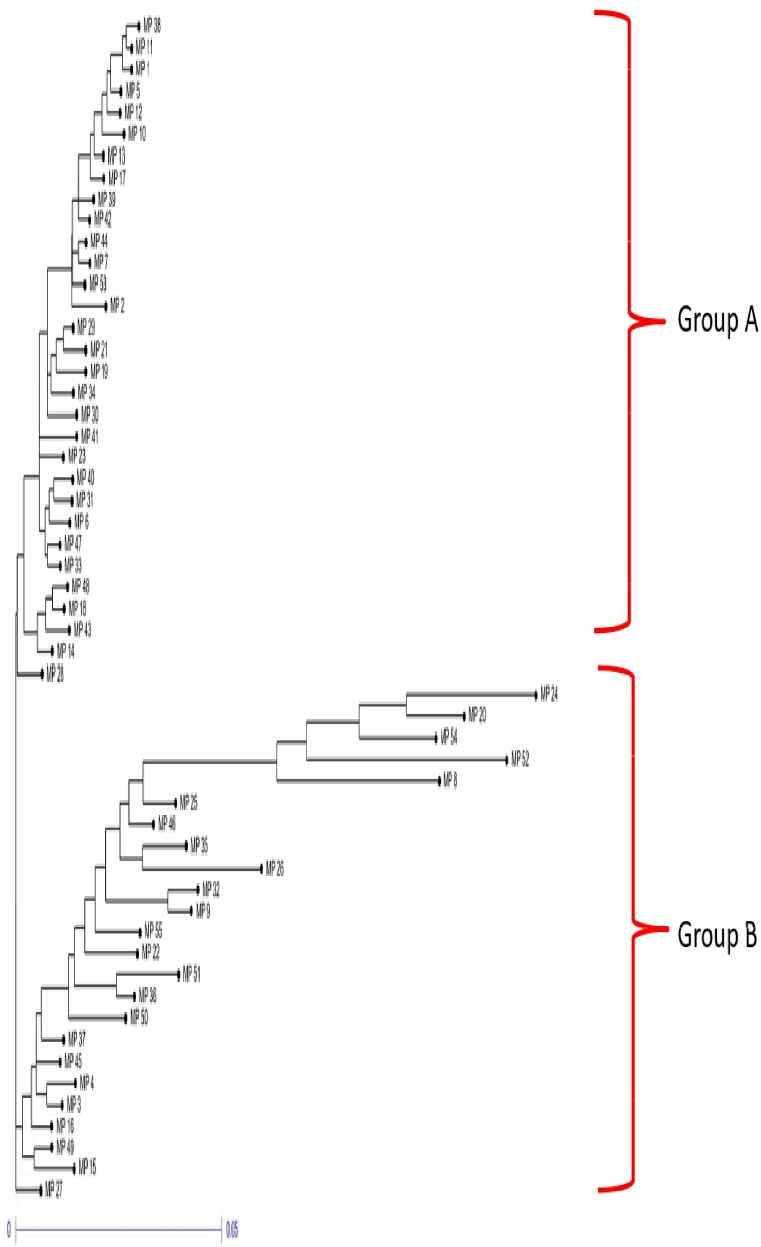
A phylogenetic tree employing the neighbor-joining (NJ) method was constructed for 55 *M. phaseolina* isolates (MP 1 to MP 55), considering 15 morpho-cultural and 3 pathogenic traits. Isolate numbers are depicted on the branch termini. To assess branch robustness, bootstrap analysis with 1000 replicates was performed. Detailed information regarding the isolates can be found in Table 1.

Cluster analysis was conducted on 55 isolates using 15 morpho-cultural and 3 pathogenic characters. The neighbor-joining cluster analysis method with 1000 bootstrap values formed two major groups: Group A and Group B (Fig. 4). Group A comprised 31 isolates, while Group B consisted of 23 isolates. Notably, one isolate, MP-27 (Pune), stood out distinctly from the rest, indicating the distinctive nature of the isolates over the remaining isolates. Interestingly, isolates tended to cluster together regardless of their agro-climatic zones. Group A includes isolates from all agro-climatic zones, while Group B exhibits greater diversity, as indicated by higher Euclidean distances among its isolates. Within Group A, isolate MP-28 (Amravati) was particularly distinct compared to the others. Group A predominantly comprises most isolates collected from central India (Western Plateau and Hills region and Central Plateau and Hills region). Interestingly, Group B contained isolates that exhibited higher virulence levels than those in Group A, including the most virulent isolate, MP-51 (Medziphema).

### 3.4 Statistical analysis of morpho-cultural and pathogenic characters of *M. phaseolina* isolates

The descriptive statistics were performed with 15 morpho-cultural and 3 pathogenic characters (Table S6). It showed that among cultural characters, radial growth at 2 days and upper surface colour of culture exhibited the maximum standard deviation. Similarly, among morphological characters, the number of sclerotial exhibited the maximum standard deviation, and among pathogenic characters, AUDPC on JS 20–98 exhibited the maximum standard deviation. This suggested that these characteristics can efficiently characterize morphological variability in *M. phaseolina.*

### 3.5 Molecular variability of *M. phaseolina* isolates

**Fig. 5:**
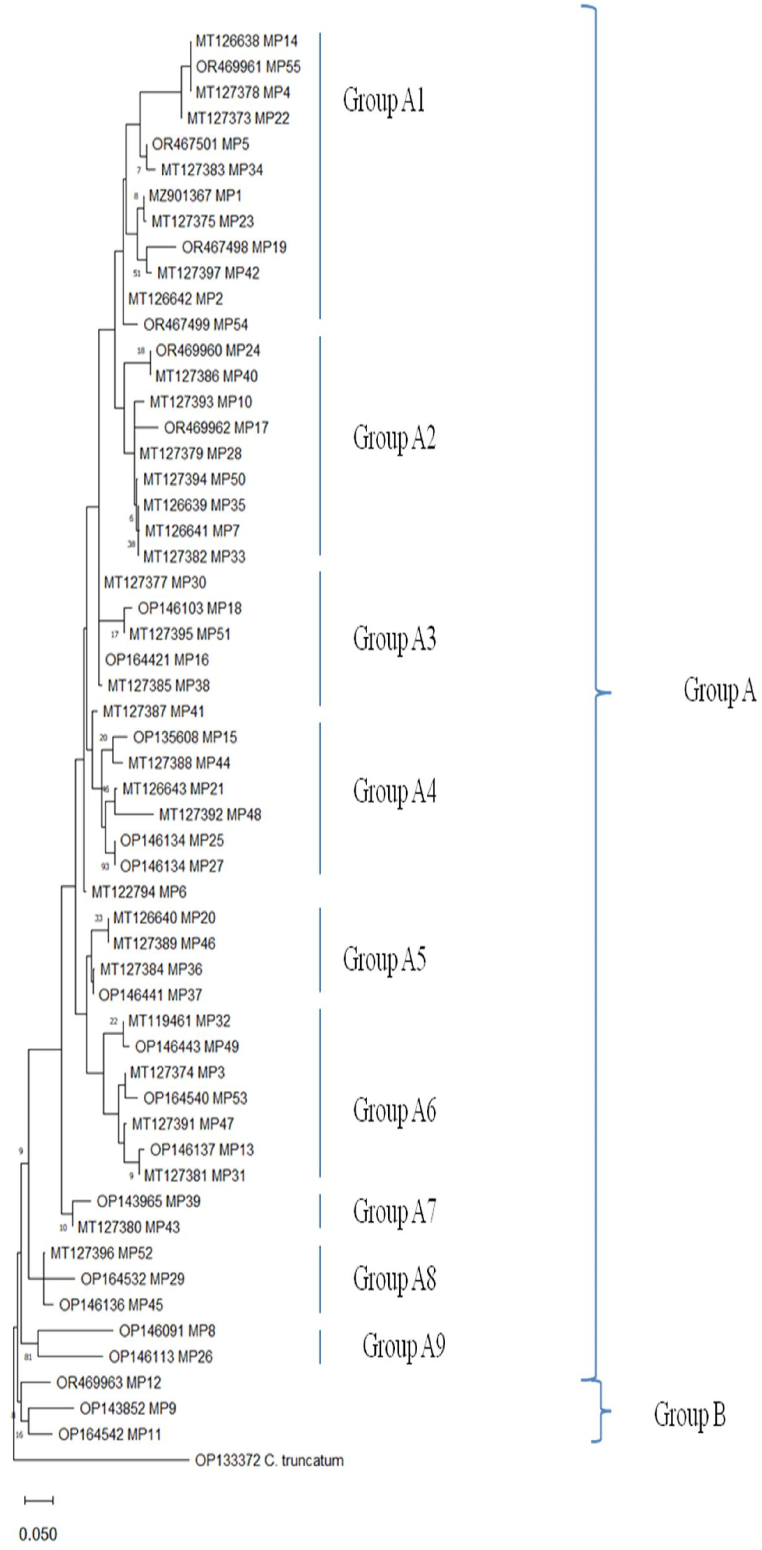
Phylogenetic tree showing phylogenetic relationships for *Macrophomina phseolina* isolates using rDNA ITS sequences. Detailed information regarding the isolates can be found in Table 1. Nucleotide sequence alignment was performed using CLUSTALW. Phylogenetic distances were determined based on the Kimura 2-parameter nucleotide substitution model. Values of Bootstrap were >50 %, calculated from 1000 replicates, are indicated alongside the branches. The scale bar indicates a single nucleotide substitution for every 100 base positions.

Phylogenetic analysis of aligned ITS sequences of *M. phaseolina* grouped 55 isolates into two groups (Fig. 5). Group A consists of 52 isolates, whereas Group B consists of 3 isolates collected from the central plateau and hills region agro-climatic region of India. Interestingly, isolates tended to cluster together regardless of their agro-climatic zones. Most of the isolates didn't show much genetic variation as they showed 99.00–100 % similarity. Among 55 isolates, 42 showed molecular variations, as depicted in the phylogeny tree.

Interestingly, the most virulent isolate, MP-51, was cluster one of the least virulent isolate, MP-18, which was completely different with respect to morpho-cultural and pathogenic characters; this result was also confirmed by cluster analysis as they grouped in different groups. Similarly, other highly virulent isolates were clustered with those isolates, which exhibited major differences in morpho-cultural and pathogenic characteristics. Genetically similar isolates, such as MP-55, MP-4, and MP-14, also clustered in different groups in cluster analysis; a similar trend was also observed with other genetically similar isolates.

The final alignment of sequences revealed 919 positions with 323/919 conserved sites, 562/919 variable sites, 237/919 parsimony informative sites, and 311/919 singleton sites. The average frequencies of four nucleotides across all the samples were A = 23.40 %, T = 24.80 %, C = 26.40 %, and G = 25.40 %. The average G + C content was measured as 51.80 %, with the maximum G + C content measured as 54.46 % in MP-29 (Amravati), and the minimum G + C content was measured as 46.52 % in MP-19 (Jabalpur). As expected, average transitional pairs (si = 10) were more prevailing than transversional pairs (sv = 12), with an average ratio of 0.80. Pair-wise, genetic distance values and evolutionary divergence of sequences based on the Kimura 2-parameter are provided in Table S7. Among major soybean-growing states, Maharashtra and Madhya Pradesh showed more divergent isolates (0.046), and Karnataka and Rajasthan showed the least divergent isolates (0.027). Most evolutionary divergent isolates were found in Bihar and Tamil Nadu (0.170), followed by Bihar to Nagaland (0.124).

## 4 Discussion

Charcoal rot caused by *M. Phaseolina* significantly threatens soybean cultivation in India and worldwide. Central India (Madhya Pradesh, Maharashtra, and Rajasthan), contributing more than 90 % of soybean area and production [3,53], was severely affected by charcoal rot disease. The increased disease pressure may be attributed to an enhanced drought period, a lack of genetically resistant genotypes, mono-cropping of susceptible cultivars (JS 95-60 and JS 335), and insufficient attentiveness to disease management practices among farmers.

A total of 55 isolates were derived from eight distinct agroecological zones associated with 13 states and two union territories of the soybean cultivation area of India in the present study. Most collections (n = 38 isolates) were obtained from Central India. They were subjected to characterization for morpho-cultural and pathogenic variability. Even though previous researchers have addressed morpho-cultural and pathogenic profiles from specific regions, there is still a lack of knowledge about the distribution and occurrence of the most prevalent *M. phaseolina* in India's major soybean growing area.

Our study showed that, the tested isolates also showed significant variability for radial growth, sclerotial characters, hyphal width, and cell size. Morpho-cultural variation in *M. phaseolina* may result from soil physical, chemical, and biological properties such as soil types, temperature, moisture, and microbiome present in different agroecological zones of India [30]. Morpho-cultural variation in *M. phaseolina* has also been reported by various researchers regarding radial growth, colour, and sclerotial size on different crops [27,28,32,33] which accords with our finding. Similarly, morpho-cultural variation in *M. phaseolina* was observed in different isolates collected from different crops, plant parts of the same host, and countries [29,32,33,54,55].

Our study showed that all the isolates showed pathogenic reactions towards soybean plants and huge pathogenic variability was observed among the isolates. We also identified several regional specific virulent isolates MP-51, MP-36, MP-34, MP-21, MP-52, MP-8, MP-24 (Mean AUDPC >70.0). The pathogenic variation in *M. phaseolina* has also been reported by various researchers on different crops [7,27,28,32,33,55]. This pathogenic variability is governed by ability to produce toxins and enzymes against host [8,34]. Additionally, researchers also identified that the pathogenic variability may be influenced by environmental factors, host-pathogen interactions, or the presence of specific virulence genes [3,6,8].

Our results indicated that the isolates collected from the tropical region of India (MP- 51), which has 25 °C to 33 °C average Kharif temperature, colonize more quickly towards the host compared to most of the isolates collected from the semi-arid region of India, which is having average temperature 24 °C to 41 °C in Kharif season [16]. The previous researchers also concluded that isolates adapted to lower temperatures (30 °C) were more virulent than isolates adapted to higher temperatures (40 °C) [32]. A temperature around 30 °C was most favourable growth conditions for *M. phaseolina*, allowing effectively establish host pathogen relationship by producing toxins and enzymes and activate virulence-related genes [6,8,32]. This outcome may also be linked to the climatic conditions maintained during the glasshouse experiment, which were more favourable for isolates adapted to lower temperatures (30 °C) compared to those adapted to higher temperatures (40 °C). The previous researchers also supported this finding [32]. Additionally, lower temperature (30 °C) enhanced aggressiveness of *M. phaseolina* towards the host by weakening the defense mechanism of host and allowing pathogen to invade host effectively [56].

Our results indicated that the isolates collected from central India, which contributes most of India's soybean area, have most of the highly virulent isolates (MP-8, MP-21, M P-24, and MP-36) as well as least virulent isolates (n = 12, AUDPC<25). In addition, other highly virulent isolates (MP-51, MP-34, and MP-52) were found across various agroecological zones of India, even in regions with limited soybean cultivation. The severity of charcoal rot disease in soybeans is linked with abiotic stresses (heat and drought), particularly in the reproductive phase [14]. The virulence of pathogen is driven by local adaptations within different pathogen populations, as well as by various genetic and environmental factors that influence pathogen aggressiveness [57]. Pathogen virulence can also shift as a result of the selection of diverse pathogen phenotypes in human-modified agro-ecosystems [9]. In the present study most of isolates collected from central India have mono-cropping of soybean from last three decades [16]. Extended mono-cropping may exert selective pressure on pathogen populations, potentially leading to population divergence. This could result in altered ecological requirements and the parallel development of partial host specialization [9]. As a result, we identified both highly virulent and least virulent isolates from central India.

Besides climatic conditions, introduction of pathogens through seed was also an important factor for pathogenic variability [46]. Our study revealed that the oldest cultivated area of soybean in India, particularly the northern hill region and northeastern hill region, served as the first entry point for soybean introduction to India through Indonesian traders from China [16], have more virulent isolates as compared to central India, where soybean was introduced from USA [16].

Our results also indicated that the pathogen's virulence did not only depend on climatic conditions. Cluster analysis for virulence characters did not clearly separate isolates collected from diverse climates, as suggested in several findings [30,31,55]. In contrast, clear-cut separation was observed in the virulence of *M. phaseolina* isolates collected from southern and northern states of the USA [32] and Mexican and non-Maxican isolates [56]. Pathogenic differentiation is also separated in *M. phaseolina* isolates collected from arid and tropical regions [58]. The isolates with lower virulence were clustered in cluster III, whereas isolates with higher virulence were grouped separately in clusters I and II. These results agreed with the Pearson correlation coefficient as indicated by the significantly higher positive correlation of the virulence of isolates (Supp Fig. 1), as suggested in several findings [59,60].

Our results showed a significant difference for G, I, and G × I. The significant G × I interaction concluded that virulence in isolates and resistance in varieties varied with the environment. Our results also indicated that the contribution of virulence of isolates (56.30 %) and genotypic variance of varieties (36.79 %) was more than the G × I interaction (4.96 %), indicating that most of the variation for disease reaction was genetic [6]. Similarly, earlier researchers also reported that in chickpea and pigeon peas wilt diseases the genotypic variation contributed more than G × I variance [59,60]. In contrast, our previous study on soybean anthracnose found that G × I contributed more than G and I [46].

Our study revealed that the performance of any genotype in specific environment was significantly affected by G × I interaction, indicating the disease reaction in genotypes depended on the interaction of virulence of the pathogen and resistance of the genotype [2,3,33,61]. For example, highly discriminative isolates MP-52 expressed more virulence towards variety Shivalik and less virulence towards variety JS 20–98. Similarly, another highly discriminative isolate, MP-50, expressed more virulence towards variety JS 20–98 and less virulence towards other varieties, Shivalik and JS 95-60. Interestingly, the most virulent isolate, MP-51, can be known in all three soybean varieties. Other highly virulence isolates, MP-8, MP-24, and MP-36, also showed more virulence towards JS 20–98 than towards other varieties. The isolates were also adapted to the specific genetic composition of soybean varieties as the genetic composition of soybean varieties differed from the central part of India to the northern hill region of India, which could be another cause of significant G × I interaction [16].

More distance to the centre of GGE biplots and positive PC1 score revealed the high level of virulence and strong discriminative ability of isolates [52,62]. For example, our study revealed that MP-50 and MP-52 showed a higher distance to the centre of GGE biplots and a high PC1 score supported by a high level of disease expression and highly discriminative than other isolates. This variation arises from the climate of a specific location, the genetic composition of varieties, and their interaction [59,60].

The present studies did not conclude any relationship between most of morpho-cultural and pathogenic characters of 55 isolates of *M. phaseolina*, these results agreed with PCA and statistics analysis. Morpho-cultural characters aerial growth (Pearson correlation coefficient (P) = 0.37∗∗ for JS 95-60, 0.29∗ for Shivalik and 0.39∗∗ for JS 20–98) and colony surface (P = 0.34∗ for JS 95-60, 0.31∗ for JS 20–98) (Supp Fig. 1), showed significant correlation with pathogenic characters of the isolates. However, the low correlation value indicated a weak association between morpho-cultural and pathogenic traits, a finding that aligns with the results of PCA and statistical analysis. Several researchers have not found an association between morpho-cultural characters and virulence characters [27,30,31,56,55]. Contradictory to that, a close association was also found between virulence and growth of fungus [28,63], number of microsclerotia [64,65] and colony colour of pathogen's [66]. The absence of correlation between morpho-cultural and pathogenic characters highlights the complexity of factors influencing the virulence of the pathogens. Morpho-cultural traits are often influenced by climatic conditions and may serve as adaptive responses to specific agro-climatic zones rather than being reliable indicators of pathogen virulence [3]. Virulence in *M. phaseolina* is governed by production of toxin and enzymes, rather than morpho-cultural characters [8,34]. The relationship between morpho-cultural traits and pathogen virulence is often influenced by the pathogen's genetic diversity in genomic regions that are unrelated to these traits. Additionally, research also identified that the variability in pathogenicity among isolates of *M. phaseolina* may be influenced by environmental factors, host-pathogen interactions, virulence factors, epigenetic modifications, or presence of specific virulence genes, rather than morphological characters [3,6,8]. Future studies focusing on functional genomic analyses, such as the role of specific effector proteins or secondary metabolite biosynthesis pathways, could further elucidate the determinants of pathogenicity.

Several researchers have found an association between virulence and fungus growth [28,63]. Contradictory, we identified that most of the highly virulent isolates displayed a range of radial growth types, including excellent growth (MP-8, MP-21, and MP-34), moderate growth (MP-51), good growth (MP-36 and MP-52), and slow growth (MP-24). In accord to that several researchers also did not find any association between virulence and the fungal growth [30,31]. This indicates that the virulence ability of *M. phaseolina* is not solely dependent on its capability to proliferate rapidly, but expected involves a complex interaction of genetic and biochemical factors that regulate establishment of host parasitic relationship and colonization [8,34].

Similarly, among the highly virulent isolates, most of the isolates produced medium-sized microsclerotia (>100–200 μm in length and width) except MP-8, which produced microsclerotia of smaller size. The isolates exhibited low virulence producing different size of microsclerotia. Similar trend was observed with number of microsclerotia, as most of the highly virulent isolates produced medium range (0.5 × 10^6^/cm^2^ to 0.3 × 10^6^/cm^2^) of microsclerotia. However, isolate MP-38, which displayed medium virulence, produced the highest microsclerotial density at 11 × 10⁶/cm^2^. Our study showed that the virulence of the isolates was not dependent on the size and number of microsclerotia, as suggested in several findings [27,30,56]. In contrast, researchers suggested that higher amount of microsclerotia is more likely to show higher virulence by quick germination and production of infectious structure and increase probability to colonize the host [64,65].

The colour of phytopathogenic fungi often accredited to melanin production, is known to be essential part of various survival strategies. It provides protection against environmental stresses, particularly UV radiation, enhanced resilience to oxidative damage, amplified antifungal tolerance ability and increased aggressiveness of pathogen [54]. Our study identified that most of the highly virulent isolates had colony colour from dark gray to light gray, which is characteristics feature of *M. phaseolina* [3]. Cell pigmentation in *M. phaseolina* is protecting the pathogen against plant immune responses and is essential for the pathogen's virulence through modulation of synthesis of cell wall-degrading enzymes [66,67]. Our study also revealed that the virulence of isolates was not dependent on colony colour (gray to black) as suggested in several findings [7,31]. In contrast, both white and coloured mycelium in *M. phaseolina* showed virulence in the host was also reported [54].

PCA further suggested that quantitative morpho-cultural and pathogenic characters were more suitable than qualitative morpho-cultural characters for *M. phaseolina* characterization and classification, as recommended by several researchers [56,58,[51], [68]]. All the tested isolates were dispersed along both horizontal and vertical axes without forming clusters, even though they belong to the same agro-ecological zones of India, indicating enormous variation between morpho-cultural and pathogenic characteristics of *M. phaseolina* as suggested in several findings [31,35].

The result of current investigation identified two major groups through cluster analysis. The cluster analysis specified relationships between the virulence of pathogens and morpho-cultural. Most of the highly virulent isolates were grouped in group B, which exhibited greater diversity among its isolates. Interestingly, isolates showed a tendency to cluster together irrespective of their agro-climatic zones [31]. In contrast, a distinct separation of *M. phaseolina* isolates was observed based on climatic conditions [32,56]. This indicated that pathogenic variations in *M. phaseolina* isolates exist within the same geographic regions.

Phylogenetical analysis of the rDNA-ITS region of fungus was a successful method for identifying the grouping of many fungi [9]. Interestingly, isolates tended to cluster together regardless of their agro-climatic zones. Forty-two isolates were found to be phylogenetically distinct through sequence analysis of rDNA-ITS. This indicated the unique nature of these isolates over the rest. Most of the isolates showed a similarity index of 99.00–100 %. The isolates showing similar morpho-cultural and pathogenic characters clustered together and vice versa. Genetically similar isolates also clustered in different groups in cluster analysis. The isolates more closely associated within a sub-group share a close resemblance, perhaps due to a common gene pool. Sequencing of a huge collection of isolates from several hosts is required to characterize the group [9]. Similarly, other researchers also identified that isolates collected from the same agro-climatic zones were not clustered [35,36]. Our study revealed, limited genetic diversity in rDNA-ITS region of *M. phaseolina*, despite having huge morpho-cultural and pathogenic variability. Limited rDNA-ITS diversity in *M. phaseolina* has been suggested in several findings and may be credited to the conserved nature of the ITS region as it has critical role in rRNA processing, which may not capture the full extent of genetic variation within the species [35,36]. However, *M. phaseolina* shows significant genetic diversity in other genomic regions rather than ITS, predominantly those regions linked to pathogenicity and adaptation to various climatic condition [34]. This adaptability underscores the importance of exploring other genomic markers for a deeper understanding of its genetic diversity. The adaptability of *M. phaseolina* to various agro-climatic zones may be influenced by other mechanisms such as horizontal gene transfer, epigenetic changes, or genomic plasticity in non-coding regions, rather than being solely influenced by the conserved rDNA-ITS region [34].

This is the first study from India to identify morpho-cultural, pathogenic and molecular variation in *M. phaseolina* causing soybean charcoal rot. This study highlights the notable variations in morphology, culture, and pathogenicity of *M. phaseolina* across different agro-ecological zones. This can be applied in disease-resistance breeding programs to identify stable and resistant genotypes through multi-environmental evaluation of genotypes. Our study identified location specific highly virulent and discriminative isolates which will be useful for screening genotypes and identifying quantitative trait loci (QTL) linked to soybean charcoal rot. This will allow mapping in the soybean genome, as, to date, no effective QTL for soybean charcoal rot has been reported from India. Therefore, further investigations are needed to screen genotypes to identify stable resistance sources for effective management of soybean charcoal rot disease. These findings highlight the significance of accounting for environmental factors, particularly temperature, when developing management strategies adapted to specific environmental conditions.

## 5 Conclusions

Our study highlights significant diversity in the morpho-cultural and pathogenic variability of *M. phaseolina*, but limited diversity in the rDNA-ITS region. These findings highlight the importance of exploring genetic markers beyond ITS to gain a more comprehensive understanding of pathogen diversity. We did not find any association between the virulence and morpho-cultural characteristics of the pathogen. The differentiation of isolates based on agroecological zone or any association between virulence and genetic structures of the isolates was not observed. Our study suggested that quantitative morpho-cultural and pathogenic characters were more suitable for *M. phaseolina* characterization and classification than qualitative morpho-cultural characters. One of the significant aspects of the present study resides in the result that the isolates adapted to low temperatures are more virulent than isolates adapted to higher temperatures. This attribute showed that the performance of any genotype in specific environments was significantly affected by G × I interaction and virulence of the isolates. These findings suggest marker-assisted selection (MAS) breeding strategies that incorporate resistance traits from specific cultivars, prioritize screening under environmentally relevant conditions such as temperature, and utilize local isolates for accurate germplasm and variety evaluation. Additionally, modified disease management strategies, including integrating resistant germplasm lines and applying environment-specific cultural or chemical controls, are recommended to target highly virulent isolates. Multi-environment trials and genomic tools like quantitative trait loci (QTL) mapping can further enhance the development of durable resistance in soybean cultivars, ensuring effective management of charcoal rot disease. Our study also identified highly virulent (MP-51) and highly discriminative (MP-50 and MP-52) isolates from various agroecological regions of India. These isolates will be useful for screening genotypes and identifying quantitative trait loci (QTL) linked to soybean charcoal rot. Additional research should be focussed on identifying specific temperature-regulated virulence factors, which may be utilized in breeding programs aimed at development of charcoal rot resistant variety to the most virulent isolates. Incorporating the current findings into breeding programs and disease management strategies will strengthen crop protection across soybean growing agro-ecological zones of India.

## CRediT authorship contribution statement

**Laxman Singh Rajput:** Writing – original draft, Investigation. **Sanjeev Kumar:** Methodology. **Kriti Pathak:** Investigation. **Palak Acharya:** Investigation. **Divyanshu Goswami:** Investigation. **Vennampally Nataraj:** Supervision, Investigation. **Maranna Shivakumar:** Resources. **Hemant Singh Maheshwari:** Writing – review & editing. **Saloni Mandloi:** Validation. **Sapna Jaiswal:** Formal analysis. **Asha Yadav:** Visualization. **Raksha Vishwakarma:** Data curation.

## Ethics approval

All authors read and comply with the statements mentioned in “Elsevier's Publishing Ethics Policy”.

## Data availability statement

The datasets analyzed during the current study are available from the corresponding author upon reasonable request. The Gene sequence datasets generated during the current study are available in the NCBI repository (https://www.ncbi.nlm.nih.gov/nuccore), with the given Accession number maintained in the manuscript).

## Declaration of competing interest

The authors declare the following financial interests/personal relationships which may be considered as potential competing interests:Hemant S. Maheshwari reports article publishing charges was provided by The University of Groningen, the Netherlands. If there are other authors, they declare that they have no known competing financial interests or personal relationships that could have appeared to influence the work reported in this paper.

## Figures and Tables

**figs1:**
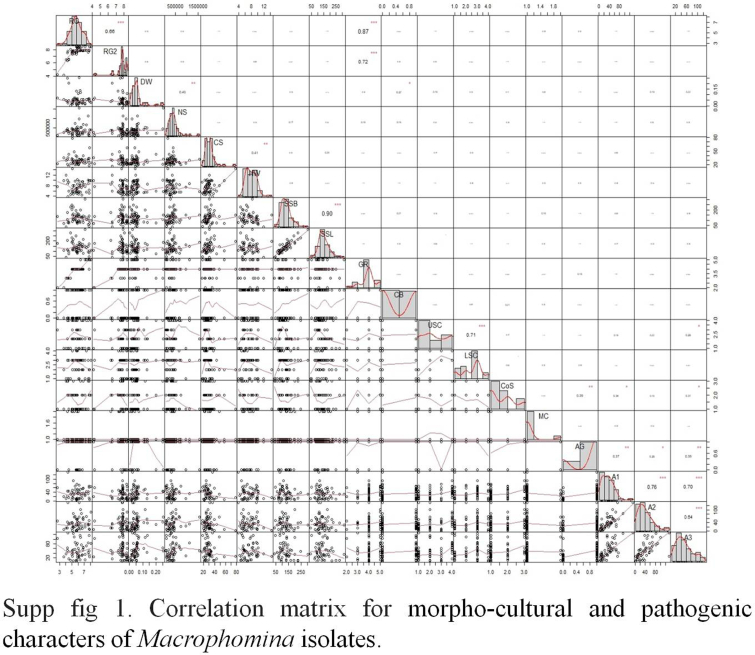
Correlation matrix for morpho-cultural and pathogenic characters of *M.* phaseolina isolates

**figs2:**
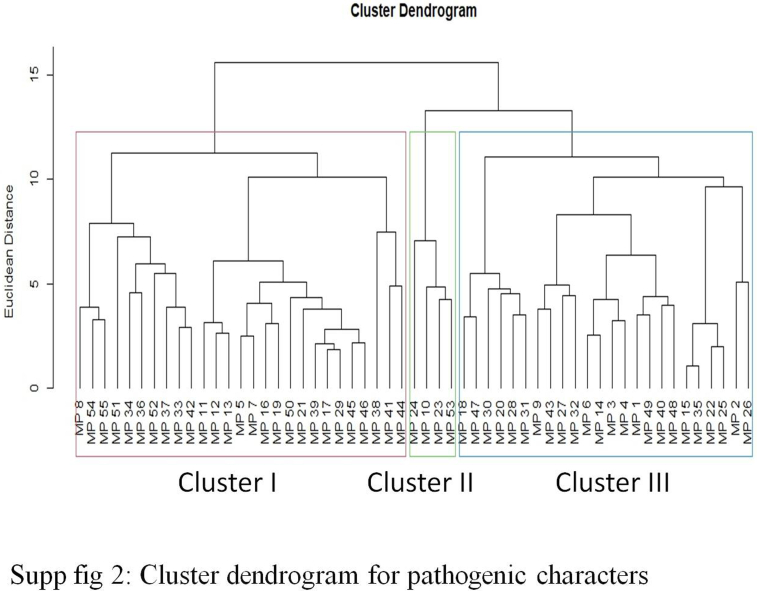
Cluster dendrogram for pathogenic characters of *M. phaseolina* isolates
