# Supplementary material for: Morpho-cultural, pathogenic, and genetic characterization of Indian isolates of Macrophomina phaseolina causing charcoal rot in soybean
Source: Heliyon. 2025 Jan 17;11(2):e42035. doi: 10.1016/j.heliyon.2025.e42035 (PMC11787666; doi:10.1016/j.heliyon.2025.e42035)
Supplement: Multimedia component 1 [file mmc1.docx]

Table S1: Cultural characteristics of *M. phaseolina* isolates on PDA at 25^0^C

| Isolates | Radial  growth  2 days | Radial  growth  3 days | Growth rate | Upper surface  colour | Lower surface  colour | Colony  Surface | Margins of  colony | Aerial growth | Constriction  at base | Dry weight  (grams) |
| --- | --- | --- | --- | --- | --- | --- | --- | --- | --- | --- |
| MP 1 | 6.20h-l | 8.30 ab | Good | Light grey | Light grey | Compact | Regular | Absent | Present | 0.020a |
| MP 2 | 7.85ab | 8.40 a | Excellent | Grey | Grey | Fluffy | Regular | Present | Absent | 0.038a |
| MP 3 | 6.60f-h | 8.40 a | Good | Light grey | Grey | Fluffy | Regular | Present | Present | 0.028a |
| MP 4 | 7.80ab | 7.90 ef | Good | Light grey | Light grey | Fluffy | Regular | Present | Present | 0.065a |
| MP 5 | 5.53n-q | 7.80 fg | Good | Dark grey | Black | Fluffy | Regular | Present | Present | 0.068a |
| MP 6 | 7.15 de | 8.40 a | Excellent | Light grey | Light grey | Compact | Regular | Present | Absent | 0.014a |
| MP 7 | 6.05j-m | 8.40 a | Good | Black | Black | Compact | Regular | Present | Present | 0.108a |
| MP 8 | 7.80 ab | 7.90 ef | Excellent | Grey | Light grey | Cottony | Regular | Present | Absent | 0.057a |
| MP 9 | 4.80 st | 8.08 cd | Moderate | Light grey | Light grey | Fluffy | Regular | Present | Present | 0.002a |
| MP 10 | 4.05vw | 6.50 no | Moderate | Light grey | Light grey | Cottony | Regular | Present | Present | 0.047a |
| MP 11 | 7.65a-c | 7.90 ef | Excellent | Dark grey | Black | Compact | Regular | Present | Absent | 0.059a |
| MP 12 | 6.80ef | 7.90 ef | Good | Dark grey | Black | Compact | Regular | Present | Absent | 0.056a |
| MP 13 | 7.35cd | 7.90 ef | Excellent | Dark grey | Dark grey | Compact | Regular | Present | Absent | 0.057a |
| MP 14 | 6.08i-m | 8.35 a | Good | Light grey | Light grey | Compact | Regular | Present | Absent | 0.040a |
| MP 15 | 5.70 m-o | 8.40 a | Good | Black | Dark grey | Compact | Regular | Absent | Absent | 0.015a |
| MP 16 | 5.15q-s | 7.80 fg | Good | Dark grey | Black | Compact | Regular | Present | Absent | 0.057a |
| MP 17 | 5.90 k-n | 7.80 fg | Good | Dark grey | Black | Fluffy | Regular | Present | Present | 0.058a |
| MP 18 | 5.80 l-o | 8.00 de | Good | Black | black | Fluffy | Irregular | Absent | Absent | 0.003a |
| MP 19 | 5.80 l-o | 8.35 a | Good | Black | Dark grey | Compact | Regular | Present | Absent | 0.028a |
| MP 20 | 5.05 rs | 7.33 lm | Good | Black | Dark grey | Fluffy | Irregular | Present | Present | 0.015a |
| MP 21 | 8.08 a | 8.40 a | Excellent | Dark grey | Black | Fluffy | Regular | Present | Present | 0.030a |
| MP 22 | 6.33 g-k | 8.28 ab | Good | Black | Black | Compact | Regular | Absent | Absent | 0.035a |
| MP 23 | 3.65 w | 4.15 p | Slow | Light grey | Grey | Compact | Regular | Present | Absent | 0.054a |
| MP 24 | 2.68 x | 4.25 p | Slow | Black | Black | Compact | Regular | Absent | Absent | 0.067a |
| MP 25 | 5.88 l-n | 8.18 bc | Good | Black | Black | Compact | Regular | Absent | Absent | 0.065a |
| MP 26 | 5.05 rs | 7.70 g-i | Good | Grey | Grey | Compact | Regular | Present | Absent | 0.043a |
| MP 27 | 5.00 rs | 7.70 g-i | Good | Dark grey | Black | Compact | Regular | Present | Absent | 0.029a |
| MP 28 | 4.30 uv | 6.35 o | Moderate | Grey | Black | Fluffy | Irregular | Present | Absent | 0.039a |
| MP 29 | 5.50 n-q | 7.80 fg | Good | Dark grey | Black | Compact | Regular | Present | Present | 0.057a |
| MP 30 | 5.40 o-r | 7.40 k-m | Good | Grey | Grey | Fluffy | Irregular | Present | Present | 0.072a |
| MP 31 | 5.00 rs | 7.45 j-l | Good | Grey | Black | Fluffy | Irregular | Present | Present | 0.018a |
| MP 32 | 7.15 de | 7.90 ef | Excellent | Grey | Black | Fluffy | Regular | Present | Present | 0.022a |
| MP 33 | 5.20 q-s | 7.63 hi | Good | Grey | Black | Fluffy | Regular | Present | Absent | 0.041a |
| MP 34 | 6.35 g-j | 7.90 ef | Excellent | Grey | Black | Cottony | Regular | Present | Present | 0.118a |
| MP 35 | 5.90k-n | 8.40 a | Good | Black | Dark grey | Compact | Regular | Absent | Absent | 0.005a |
| MP 36 | 5.18 q-s | 7.80 fg | Good | Dark grey | Black | Cottony | Regular | Present | Absent | 0.040a |
| MP 37 | 6.83 ef | 7.80 fg | Good | Light grey | Grey | Cottony | Regular | Present | Absent | 0.050a |
| MP 38 | 6.20 h-l | 7.80 fg | Good | Grey | Black | Compact | Regular | Absent | Present | 0.255a |
| MP 39 | 6.50 f-i | 7.90 ef | Good | Dark grey | Black | Compact | Regular | Present | Present | 0.055a |
| MP 40 | 5.83 l-o | 7.60 h-j | Good | Grey | Dark grey | Compact | Regular | Absent | Present | 0.054a |
| MP 41 | 4.46 t-v | 6.53 n | Moderate | Dark grey | Black | Fluffy | Regular | Absent | Present | 0.202a |
| MP 42 | 5.70 m-o | 7.28 m | Good | Grey | Grey | Fluffy | Regular | Present | Absent | 0.130a |
| MP 43 | 5.25 p-r | 7.75 f-h | Good | Grey | Black | Cottony | Regular | Present | Present | 0.052a |
| MP 44 | 4.26 uv | 7.54 i-k | Moderate | Dark grey | Black | Cottony | Regular | Present | Present | 0.250a |
| MP 45 | 5.70 m-o | 7.60 h-j | Good | Dark grey | Black | Fluffy | Regular | Present | Present | 0.054a |
| MP 46 | 6.73 e-g | 7.80 fg | Good | Dark grey | Black | Fluffy | Regular | Present | Present | 0.031a |
| MP 47 | 6.04 j-m | 7.80 fg | Good | Grey | Grey | Compact | Irregular | Absent | Present | 0.019a |
| MP 48 | 5.66 m-p | 7.60 h-j | Good | Light grey | Grey | Compact | Regular | Absent | Absent | 0.020a |
| MP 49 | 6.70 fg | 7.80 fg | Good | Grey | Grey | Compact | Regular | Absent | Present | 0.022a |
| MP 50 | 6.33 g-k | 7.80 fg | Good | Grey | Black | Compact | Regular | Present | Present | 0.137a |
| MP 51 | 4.34 uv | 7.75 f-h | Moderate | Light grey | Light grey | Cottony | Regular | Present | Absent | 0.023a |
| MP 52 | 6.15 i-l | 7.80 fg | Good | Grey | Grey | Compact | Regular | Present | Absent | 0.033a |
| MP 53 | 4.50 tu | 6.50 no | Moderate | Light grey | Light grey | Compact | Regular | Present | Absent | 0.048a |
| MP 54 | 7.80 ab | 7.80 fg | Excellent | Light grey | Grey | Cottony | Regular | Present | Present | 0.048a |
| MP 55 | 7.60 bc | 7.60 h-j | Excellent | Light grey | Light grey | Cottony | Regular | Present | Absent | 0.055a |

Different superscript letters are statistically different at p < 0.05 based on Fisher’s protected Least Significant Difference test.

Table S2: Morphological characteristics of *M. phaseolina* isolates on PDA at 25^0^C

| Isolates | Number of sclerotial (cm^2^) | Length of micro-sclerotia  (µm) | Range | Width of micro-sclerotia (µm) | Range | Mycelial  cell size  (µm) | Range | Hyphal  width  (µm) | Range |
| --- | --- | --- | --- | --- | --- | --- | --- | --- | --- |
| MP 1 | 967500 bc | 161.93 f-l | 117.6-239.6 | 148.65 g-j | 110.6-165.2 | 23.37 k-t | 17.4-27.6 | 7.36 g-n | 5.7-9.6 |
| MP 2 | 397500 e-l | 105.42 n-t | 66.7-153.9 | 68.42 w-z | 30.6-98.8 | 57.60 a | 23.8-78.7 | 9.89 cd | 6.7-13.1 |
| MP 3 | 228750 h-n | 148.68 h-n | 122.9-189.14 | 132.71 i-o | 65.1-170.6 | 31.11 e-o | 21.1-40.6 | 4.33 tu | 3.6-5.3 |
| MP 4 | 236250 g-n | 173.81 e-j | 90.9-296.0 | 139.27 h-l | 117.7-156.7 | 34.73 d-i | 23.9-48.5 | 9.83 cd | 6.9-13.2 |
| MP 5 | 746250 cd | 113.00 m-t | 71.9-173.39 | 104.51 m-u | 75.6-128.9 | 20.80 q-t | 13.0-33.3 | 5.62 n-t | 3.4-7.3 |
| MP 6 | 393750 e-l | 147.17 h-n | 110.2-183.4 | 130.75 i-p | 85.5-155.8 | 24.16 k-t | 19.1-25.9 | 7.52 f-m | 4.9-9.6 |
| MP 7 | 427500 e-k | 110.69 n-t | 86.1-139.7 | 85.84 s-x | 70.4-105.9 | 21.31 p-t | 15.8-27.2 | 5.28 q-u | 3.1-6.3 |
| MP 8 | 101250 l-n | 49.41 uv | 41.8-65.6 | 43.94 yz | 39.7-51.7 | 18.35 st | 9.9-25.1 | 5.14 q-u | 4.0-6.2 |
| MP 9 | 240000 g-n | 280.93 ab | 109.2-389.0 | 225.11 bc | 93.1-350.8 | 22.89 m-t | 16.7-29.8 | 4.52 s-u | 3.7-5.2 |
| MP 10 | 356250 f-n | 41.26 v | 29.8-68.65 | 36.48 z | 21.4-50.8 | 30.66 e-p | 16.8-48.0 | 11.94 b | 9.4-14.7 |
| MP 11 | 1128750 b | 146.83 h-n | 92.8-236.7 | 122.86 j-r | 98.0-125.1 | 30.15 e-q | 21.9-43.2 | 8.65 c-g | 8.1-9.6 |
| MP 12 | 678750 c-e | 81.61 s-v | 45.8-142.2 | 81.29 t-x | 53.2-104.8 | 25.06 i-t | 17.7-40.42 | 8.44 c-i | 6.2-12.2 |
| MP 13 | 656250 d-f | 136.94 i-p | 117.9-168.7 | 110.58 l-t | 74.2-140.0 | 26.14 h-t | 16.5-35.6 | 5.46 p-u | 3.8-7.58 |
| MP 14 | 240000 g-n | 130.56 j-r | 81.6-184.0 | 98.70 p-w | 73.8-124.6 | 19.94 r-t | 13.0-24.5 | 4.86 r-u | 4.1-6.0 |
| MP 15 | 161250 i-n | 95.36 p-t | 74.1-160.3 | 92.15 r-x | 71.6-148.5 | 21.74 n-t | 14.7-35.5 | 6.78 h-q | 4.2-8.5 |
| MP 16 | 221250 h-n | 123.39 k-s | 101.5-164.4 | 115.15 k-s | 95.4-127.2 | 27.67 g-s | 19.1-34.5 | 7.98 e-k | 5.4-9.9 |
| MP 17 | 543750 d-g | 90.86 q-u | 53.8-121.9 | 86.03 s-x | 56.1-106.2 | 27.19 g-s | 21.5-37.0 | 8.62 c-g | 4.3-10.6 |
| MP 18 | 292500 g-n | 157.17 g-m | 110.6-208.8 | 133.37 i-n | 93.8-188.0 | 21.57 o-t | 17.5-23.8 | 5.56 o-u | 4.5-6.9 |
| MP 19 | 333750 g-n | 94.09 p-u | 84.1-119.9 | 81.90 t-x | 72.0-95.2 | 24.98 i-t | 18.5-31.8 | 6.70 i-q | 4.3-11.2 |
| MP 20 | 75000 n | 116.88 l-t | 81.1-174.8 | 87.83 s-x | 58.1-106.7 | 22.37 m-t | 17.6-25.2 | 5.70 n-t | 4.2-6.9 |
| MP 21 | 382500 e-n | 112.36 m-t | 87.6-137.36 | 97.55 q-w | 87.7-114.9 | 24.60 j-t | 12.8-35.8 | 9.88 cd | 9.3-11.3 |
| MP 22 | 127500 k-n | 104.65 n-t | 74.9-160.7 | 90.27 r-x | 62.9-125.5 | 29.78 f-q | 18.3-43.3 | 8.01 e-j | 7.1-8.9 |
| MP 23 | 255000 g-n | 109.36 n-t | 47.2-165.9 | 68.97 w-z | 34.5-96.4 | 35.21 d-h | 24.6-60.8 | 9.82 cd | 7.6-11.3 |
| MP 24 | 97500 l-n | 179.76 e-i | 127.4-262.1 | 135.94 h-m | 92.33-188.0 | 23.08 l-t | 20.4-25.4 | 7.61 f-l | 6.1-9.47 |
| MP 25 | 123750 k-n | 96.55 o-t | 81.5-109.5 | 72.61 u-y | 61.3-85.2 | 26.26 g-t | 18.8-34.1 | 10.19 bc | 7.6-11.2 |
| MP 26 | 161250 i-n | 194.68 d-g | 133.9-260.3 | 152.83 g-j | 93.5-259.8 | 59.32 a | 41.6-141.8 | 14.46 a | 4.2-20.4 |
| MP 27 | 431250 e-k | 229.06 cd | 160.5-262.2 | 246.45 b | 191.2-348.8 | 33.93 d-j | 22.0-42.6 | 6.42 j-r | 4.3-8.0 |
| MP 28 | 356250 f-n | 206.72 c-f | 140.6-345.1 | 175.18 d-g | 148.7-196.4 | 31.05 e-p | 28.7-33.4 | 8.63 c-g | 7.5-10.2 |
| MP 29 | 382500 e-n | 131.84 j-r | 87.5-189.9 | 111.45 l-t | 92.3-136.2 | 28.53 f-r | 17.5-43.2 | 9.65 c-e | 6.9-11.9 |
| MP 30 | 390000 e-m | 142.34 h-n | 89.3-170.0 | 134.50 h-n | 98.3-167.2 | 49.01 bc | 34.7-67.5 | 7.91 e-k | 5.7-10.2 |
| MP 31 | 465000 d-i | 186.87 d-h | 126.2-272.0 | 200.85 cd | 129.6-295.9 | 31.95 e-m | 26.8-40.8 | 5.20 q-u | 4.8-5.7 |
| MP 32 | 281250 g-n | 314.65 a | 154.2-477.8 | 304.20 a | 157.0-489.7 | 30.83 e-p | 24.9-37.3 | 8.28 d-i | 7.2-9.4 |
| MP 33 | 431250 e-k | 171.39 e-j | 128.1-221.9 | 162.80 f-i | 136.6-220.4 | 31.50 e-n | 22.8-37.1 | 5.78 m-t | 3.4-8.1 |
| MP 34 | 525000 d-h | 179.58 e-i | 76.1-256.8 | 199.10 c-e | 71.9-282.4 | 27.59 g-s | 20.5-32.7 | 5.50 o-u | 3.5-8.3 |
| MP 35 | 90000 l-n | 79.72 s-v | 60.7-94.9 | 70.83 v-y | 61.9-77.9 | 23.65 k-t | 20.5-29.8 | 5.86 l-t | 2-8.1 |
| MP 36 | 301875 g-n | 116.87 l-t | 74.4-182.2 | 109.00 l-t | 85.1-140.4 | 24.87 j-t | 20.5-30.2 | 4.82 r-u | 3.89-5.8 |
| MP 37 | 333750 g-n | 216.27 c-e | 127.2-287.3 | 139.53 h-l | 95.5-190.4 | 39.62 c-e | 36.6-49.3 | 8.14 d-j | 5.0-11.6 |
| MP 38 | 1563750 a | 185.35 d-h | 99.0-352.2 | 148.78 g-j | 96.5-241.2 | 32.82 e-l | 21.4-46.7 | 5.74 n-t | 4.6-7.4 |
| MP 39 | 528750 d-h | 109.57 n-t | 68.0-135.9 | 128.28 j-q | 69.6-215.7 | 32.08 e-m | 20.2-62.3 | 7.16 g-p | 4.8-9.6 |
| MP 40 | 352500 f-n | 135.40 i-q | 82.6-202.5 | 167.22 e-h | 145.9-175.5 | 37.48 def | 23.8-46.0 | 4.93 r-u | 3.3-7.4 |
| MP 41 | 221250 h-n | 75.07 t-v | 66.7-85.9 | 74.32 u-y | 56.9-88.5 | 43.57 cd | 33.8-56.0 | 5.89 l-t | 4.6-8.1 |
| MP 42 | 506250 d-h | 120.21 l-t | 101.8-155.2 | 137.64 h-m | 95.2-179.3 | 30.09 e-q | 27.8-32.2 | 5.57 o-u | 4.5-7.1 |
| MP 43 | 371250 e-n | 214.77 c-e | 156.6-280.2 | 224.71 bc | 191.4-257.2 | 26.15 h-t | 22.5-29.4 | 6.82 h-q | 3.6-9.1 |
| MP 44 | 442500 d-j | 121.04 k-s | 65.7-205.0 | 107.95 l-t | 75.1-150.5 | 21.98 n-t | 15.8-27.9 | 10.14 c | 8.9-11.0 |
| MP 45 | 281250 g-n | 136.45 i-p | 101.2-167.2 | 146.31 g-k | 123.1-167.0 | 27.98 f-s | 23.3-32.9 | 7.08 g-p | 6.1-8.2 |
| MP 46 | 195000 i-n | 165.75 f-k | 126.0-262.5 | 102.56 n-v | 84.4-143.4 | 27.93 f -s | 22.5-31.3 | 9.47 c-e | 5.8-13.0 |
| MP 47 | 393750 e-l | 149.17 h-n | 134.0-160.0 | 161.94 f-i | 145.0-170.2 | 28.26 f-r | 22.3-38.0 | 7.24 g-o | 4.3-10.2 |
| MP 48 | 431250 e-k | 248.00 bc | 145.3-302.2 | 194.17 c-f | 148.2-272.0 | 39.73 c-e | 28.4-56.6 | 6.41 j-r | 4.6-9.2 |
| MP 49 | 238125 g-n | 141.73 h-o | 67.8-178.0 | 127.65 j-q | 78.9-167.2 | 30.84 e-p | 23.4-35.2 | 9.58 c-e | 8.2-12.1 |
| MP 50 | 236250 g-n | 162.06 f-l | 98.3-277.7 | 81.34 t-x | 64.8-103.3 | 26.28 g-t | 18.3-33.0 | 8.54 c-h | 6.6-10.2 |
| MP 51 | 322500 g-n | 109.14 n-t | 44.2-149.4 | 80.52 t-x | 45.0-123.9 | 35.96 d-g | 22.6-47.4 | 3.82 u | 3.2-4.4 |
| MP 52 | 116250 l-n | 124.72 k-s | 91.9-160.7 | 136.35 h-m | 67.3-182.5 | 30.87 e-p | 24.8-35.4 | 9.59 c-e | 6.8-12.0 |
| MP 53 | 318750 g-n | 95.21 p-t | 79.2-115.9 | 84.45 s-x | 56.3-118.8 | 17.06 t | 12.5-26.0 | 9.23 c-f | 6.6-11.2 |
| MP 54 | 82500 mn | 86.94 r-u | 71.5-115.7 | 99.94 o-w | 79.1-119.0 | 24.79 j-t | 19.7-29.1 | 6.22 k-s | 5.5-7.2 |
| MP 55 | 146250 j-n | 96.08 p-t | 51.2-120.3 | 60.63 x-z | 44.8-83.3 | 32.95 e-k | 22.9-46.1 | 10.12 c | 9.0-11.5 |

Different superscript letters are statistically different at p < 0.05 based on Fisher’s protected Least Significant Difference test.

Table S3: AUDPC of *M. phaseolina* in three different varieties in glass house condition

| Isolate number | Varieties | | | |
| --- | --- | --- | --- | --- |
|  | JS 95-60 | Shivalik | JS 20-98 | Mean |
| MP 1 | 10.06^d-g^ | 24.54^c-e^ | 27.73^f-m^ | 20.78^o-u^ |
| MP 2 | 6.23^e-g^ | 8.36^e^ | 23.85^h-m^ | 12.81^s-v^ |
| MP 3 | 24.97^c-g^ | 24.47^c-e^ | 31.43^f-m^ | 26.96^n-t^ |
| MP 4 | 13.41^d-g^ | 10.24^e^ | 32.12^f-m^ | 18.59^p-v^ |
| MP 5 | 66.59^b-f^ | 22.53^c-e^ | 56.63^c-m^ | 48.58^h-l^ |
| MP 6 | 24.19^c-g^ | 20.18^c-e^ | 11.96^lm^ | 18.78^p-v^ |
| MP 7 | 48.32^b-g^ | 40.88^b-e^ | 60.69^b-m^ | 49.96^h-k^ |
| MP 8 | 63.28^b-g^ | 49.69^b-e^ | 116.64^ab^ | 76.54^c-e^ |
| MP 9 | 30.92^c-g^ | 37.40^b-e^ | 10.24^m^ | 26.19^n-t^ |
| MP 10 | 11.06^d-g^ | 15.66^de^ | 36.74^f-m^ | 21.15^o-u^ |
| MP 11 | 33.76^b-g^ | 32.75^b-e^ | 83.37^a-g^ | 49.96^h-k^ |
| MP 12 | 36.38^b-g^ | 65.07^a-e^ | 63.99^b-m^ | 55.14^f-j^ |
| MP 13 | 43.07^b-g^ | 69.13^a-e^ | 70.38^a-l^ | 60.86^e-h^ |
| MP 14 | 7.20^e-g^ | 13.39^de^ | 21.11^j-m^ | 13.90^r-v^ |
| MP 15 | 2.44^g^ | 2.23^e^ | 5.90^m^ | 3.52^v^ |
| MP 16 | 25.31^c-g^ | 22.55^c-e^ | 39.93^f-m^ | 29.26^n-s^ |
| MP 17 | 53.28^b-g^ | 56.87^b-e^ | 80.96^a-i^ | 63.70^d-h^ |
| MP 18 | 5.82^e-g^ | 4.24^e^ | 8.26^m^ | 6.11^uv^ |
| MP 19 | 75.63^a-c^ | 56.02^b-e^ | 41.67^f-m^ | 57.77^f-i^ |
| MP 20 | 50.31^b-g^ | 38.76^b-e^ | 52.78^c-m^ | 47.28^h-m^ |
| MP 21 | 65.44^b-f^ | 89.07^a-c^ | 82.38^a-h^ | 78.96^cd^ |
| MP 22 | 11.95^d-g^ | 9.94^e^ | 10.44^m^ | 10.78^t-v^ |
| MP 23 | 18.69^c-g^ | 8.82^e^ | 24.42^g-m^ | 17.31^p-v^ |
| MP 24 | 60.18^b-g^ | 47.48^b-e^ | 102.65^a-e^ | 70.10^d-f^ |
| MP 25 | 33.13^b-g^ | 44.64^b-e^ | 31.51^f-m^ | 36.43^k-o^ |
| MP 26 | 13.91^d-g^ | 3.00^e^ | 26.99^f-m^ | 14.63^q-v^ |
| MP 27 | 37.80^b-g^ | 41.14^b-e^ | 48.62^d-m^ | 42.52^i-n^ |
| MP 28 | 22.88^c-g^ | 14.84^de^ | 52.33^c-m^ | 30.02^n-r^ |
| MP 29 | 50.34^b-g^ | 69.17^a-e^ | 85.34^a-f^ | 68.29^d-g^ |
| MP 30 | 31.99^b-g^ | 67.59^a-e^ | 109.23^a-c^ | 69.60^d-g^ |
| MP 31 | 5.18^fg^ | 13.14^de^ | 35.53^f-m^ | 17.95^p-v^ |
| MP 32 | 29.98^c-g^ | 28.76^c-e^ | 34.68^f-m^ | 31.14^m-q^ |
| MP 33 | 36.61^b-g^ | 19.29^c-e^ | 55.12^c-m^ | 37.01^k-o^ |
| MP 34 | 52.68^b-g^ | 100.81^ab^ | 107.68^a-d^ | 87.06^bc^ |
| MP 35 | 12.23^d-g^ | 7.91^e^ | 22.71^i-m^ | 14.29^r-v^ |
| MP 36 | 92.52^ab^ | 81.83^a-d^ | 125.62^a^ | 99.99^b^ |
| MP 37 | 58.57^b-g^ | 53.90^b-e^ | 62.62^b-m^ | 58.36^f-i^ |
| MP 38 | 42.46^b-g^ | 66.62^a-e^ | 50.12^c-m^ | 53.07^g-k^ |
| MP 39 | 32.69^b-g^ | 81.93^a-d^ | 75.71^a-k^ | 63.44^d-h^ |
| MP 40 | 8.08^e-g^ | 28.34^c-e^ | 8.72^m^ | 15.05^q-v^ |
| MP 41 | 13.83^d-g^ | 32.57^b-e^ | 16.44^k-m^ | 20.95^o-u^ |
| MP 42 | 11.07^d-g^ | 56.29^b-e^ | 43.98^e-m^ | 37.12^k-o^ |
| MP 43 | 13.26^d-g^ | 17.97^de^ | 7.99^m^ | 13.08^s-v^ |
| MP 44 | 45.45^b-g^ | 43.76^b-e^ | 62.79^b-m^ | 50.67^h-k^ |
| MP 45 | 40.59^b-g^ | 34.56^b-e^ | 76.37^a-j^ | 50.51^h-k^ |
| MP 46 | 55.19^b-g^ | 58.67^b-e^ | 54.50^c-m^ | 56.12^f-i^ |
| MP 47 | 32.94^b-g^ | 28.48^c-e^ | 35.00^f-m^ | 32.14^l-p^ |
| MP 48 | 8.16^e-g^ | 4.20^e^ | 9.88^m^ | 7.41^uv^ |
| MP 49 | 9.59^e-g^ | 13.02^de^ | 31.88^f-m^ | 18.16^p-v^ |
| MP 50 | 31.69^b-g^ | 37.79^b-e^ | 116.31^ab^ | 61.93^e-h^ |
| MP 51 | 131.47^a^ | 133.44^a^ | 126.69^a^ | 130.54^a^ |
| MP 52 | 71.37^a-d^ | 134.16^a^ | 26.62^f-m^ | 77.38^c-e^ |
| MP 53 | 45.52^b-g^ | 37.07^b-e^ | 45.64^e-m^ | 42.75^i-n^ |
| MP 54 | 67.32^b-e^ | 32.41^b-e^ | 50.69^c-m^ | 50.14^h-k^ |
| MP 55 | 43.25^b-g^ | 29.82^c-e^ | 43.89^e-m^ | 38.99^j-n^ |
| LSD | 61.60 | 70.38 | 59.44 | 16.85 |
| Mean | 35.82^c^ | 39.77^b^ | 50.50^a^ |  |

Different superscript letters are statistically different at p < 0.05 based on Fisher’s protected Least Significant Difference test.

Table S4: Effect of isolates (N=55) and varieties (N=3) on indicator of charcoal rot disease development

| Source of variation | Df | MSS | F value | P value |
| --- | --- | --- | --- | --- |
| Isolates | 54 | 6218.7 | 18.82 | <0.001 |
| Varieties | 2 | 9517.4 | 28.81 | <0.001 |
| Isolates × Varieties | 108 | 838.4 | 2.53 | <0.001 |
| Error | 282 | 330.3 |  | - |

Table S5: Eigenvalue variance and Variance percent for morpho-cultural and pathogenic characters of isolates of *M. phaseolina*

| Dimension | Eigenvalue variance | Percent cumulative | Variance percent |
| --- | --- | --- | --- |
| Dim.1 | 3.20 | 17.80 | 17.80 |
| Dim.2 | 2.62 | 14.54 | 32.34 |
| Dim.3 | 2.11 | 11.75 | 44.09 |
| Dim.4 | 2.06 | 11.44 | 55.53 |
| Dim.5 | 1.52 | 8.45 | 63.98 |
| Dim.6 | 1.27 | 7.04 | 71.02 |
| Dim.7 | 1.15 | 6.41 | 77.43 |
| Dim.8 | 0.83 | 4.61 | 82.04 |
| Dim.9 | 0.68 | 3.76 | 85.79 |
| Dim.10 | 0.66 | 3.67 | 89.46 |
| Dim.11 | 0.44 | 2.47 | 91.93 |
| Dim.12 | 0.36 | 1.98 | 93.91 |
| Dim.13 | 0.33 | 1.86 | 95.76 |
| Dim.14 | 0.27 | 1.50 | 97.27 |
| Dim.15 | 0.21 | 1.14 | 98.41 |
| Dim.16 | 0.15 | 0.82 | 99.22 |
| Dim.17 | 0.10 | 0.58 | 99.80 |
| Dim.18 | 0.04 | 0.20 | 100.00 |

Table S6: Descriptive statistics for morpho-cultural characteristics and pathogenic behaviour for isolates of *Rhizoctonia* from seven maize cropping zones of India

| Variable | Min. | Max. | Mean | SD | SE | CV |
| --- | --- | --- | --- | --- | --- | --- |
| Radial Growth 2 days | 2.68 | 8.08 | 5.90 | 1.15 | 0.16 | 0.20 |
| Radial Growth 3 days | 4.15 | 8.40 | 7.66 | 0.82 | 0.11 | 0.11 |
| Growth rate | 2.00 | 5.00 | 3.98 | 0.67 | 0.09 | 0.17 |
| Upper surface colour | 1.00 | 4.00 | 2.45 | 1.14 | 0.15 | 0.46 |
| Lower surface colour | 1.00 | 4.00 | 2.53 | 0.91 | 0.12 | 0.36 |
| Constriction at base | 0.00 | 1.00 | 0.49 | 0.50 | 0.07 | 1.02 |
| Colony Surface | 1.00 | 3.00 | 1.69 | 0.76 | 0.10 | 0.45 |
| Margins of colony | 1.00 | 2.00 | 1.11 | 0.31 | 0.04 | 0.28 |
| Aerial growth | 0.00 | 1.00 | 0.76 | 0.42 | 0.06 | 0.56 |
| Dry weight | 0.00 | 0.25 | 0.06 | 0.05 | 0.01 | 0.91 |
| Number of sclerotial | 75000.00 | 1563750.00 | 369272.73 | 261158.36 | 35214.59 | 0.71 |
| Width of micro-sclerotia | 36.48 | 304.20 | 124.77 | 51.63 | 6.96 | 0.41 |
| Length of micro-sclerotia | 41.26 | 314.65 | 141.01 | 53.08 | 7.16 | 0.38 |
| Mycelial cell size | 17.06 | 78.32 | 29.97 | 9.80 | 1.32 | 0.33 |
| Hyphal width | 3.82 | 14.46 | 7.45 | 2.12 | 0.29 | 0.28 |
| AUDPC on JS 95-60 | 2.44 |  | 35.82 | 25.08 | 3.38 | 0.70 |
| AUDPC on Shivalik | 2.23 | 134.16 | 39.77 | 29.90 | 4.03 | 0.75 |
| AUDPC on JS 20-98 | 5.90 | 126.69 | 50.50 | 32.57 | 4.39 | 0.64 |

Table S7: Table: Evolutionary Divergence over sequence pairs between groups in *Macrophomina phseolina* isolates using rDNA ITS sequences based on the Kimura 2-parameter model

| States | MP | TN | MH | ND | PB | JK | RJ | KR | NG | MN | BH | UP | CG | GJ | TG |
| --- | --- | --- | --- | --- | --- | --- | --- | --- | --- | --- | --- | --- | --- | --- | --- |
| TN | 0.044 |  |  |  |  |  |  |  |  |  |  |  |  |  |  |
| MH | 0.046 | 0.039 |  |  |  |  |  |  |  |  |  |  |  |  |  |
| ND | 0.037 | 0.064 | 0.033 |  |  |  |  |  |  |  |  |  |  |  |  |
| PB | 0.043 | 0.078 | 0.040 | 0.041 |  |  |  |  |  |  |  |  |  |  |  |
| JK | 0.046 | 0.050 | 0.043 | 0.034 | 0.046 |  |  |  |  |  |  |  |  |  |  |
| RJ | 0.030 | 0.035 | 0.029 | 0.019 | 0.024 | 0.016 |  |  |  |  |  |  |  |  |  |
| KR | 0.034 | 0.065 | 0.036 | 0.035 | 0.037 | 0.015 | 0.027 |  |  |  |  |  |  |  |  |
| NG | 0.032 | 0.099 | 0.032 | 0.044 | 0.060 | 0.010 | 0.024 | 0.052 |  |  |  |  |  |  |  |
| MN | 0.026 | 0.006 | 0.025 | 0.006 | 0.015 | 0.008 | 0.010 | 0.016 | 0.010 |  |  |  |  |  |  |
| BH | 0.106 | 0.170 | 0.101 | 0.124 | 0.087 | 0.075 | 0.090 | 0.090 | 0.122 | 0.093 |  |  |  |  |  |
| UP | 0.030 | 0.095 | 0.030 | 0.038 | 0.033 | 0.006 | 0.019 | 0.042 | 0.054 | 0.008 | 0.106 |  |  |  |  |
| CG | 0.029 | 0.100 | 0.030 | 0.040 | 0.048 | 0.006 | 0.023 | 0.044 | 0.088 | 0.008 | 0.112 | 0.033 |  |  |  |
| GJ | 0.030 | 0.074 | 0.031 | 0.031 | 0.049 | 0.008 | 0.019 | 0.039 | 0.062 | 0.009 | 0.110 | 0.027 | 0.056 |  |  |
| TG | 0.033 | 0.100 | 0.033 | 0.045 | 0.034 | 0.008 | 0.022 | 0.055 | 0.077 | 0.010 | 0.114 | 0.033 | 0.053 | 0.043 |  |
| CT | 0.333 | 0.353 | 0.329 | 0.345 | 0.313 | 0.342 | 0.308 | 0.307 | 0.297 | 0.298 | 0.352 | 0.293 | 0.293 | 0.295 | 0.288 |

MP= Madhya Pradesh; TN = Tamil Nadu; MH= Maharashtra; ND = New Delhi; PB=Punjab; JK= Jharkhand; RJ= Rajasthan; KR= Karnataka; NG= Nagaland; MN= Manipur; BH= Bihar; UP=Uttar Pradesh; CG=Chhattisgarh; GJ=Gujarat, TG= Telangana
